# E-cigarettes and their lone constituents induce cardiac arrhythmia and conduction defects in mice

**DOI:** 10.1038/s41467-022-33203-1

**Published:** 2022-10-25

**Authors:** Alex P. Carll, Claudia Arab, Renata Salatini, Meredith D. Miles, Matthew A. Nystoriak, Kyle L. Fulghum, Daniel W. Riggs, Gregg A. Shirk, Whitney S. Theis, Nima Talebi, Aruni Bhatnagar, Daniel J. Conklin

**Affiliations:** 1 Christina Lee Brown Envirome Institute, University of Louisville, Louisville, KY USA; 2 American Heart Association Tobacco Regulation and Addiction Center, Louisville, KY USA; 3 Diabetes and Obesity Center, University of Louisville, Louisville, KY USA; 4 Department of Physiology, School of Medicine, University of Louisville, Louisville, KY USA; 5 Center for Integrative Environmental Health Sciences, School of Medicine, University of Louisville, Louisville, KY USA; 6 Division of Environmental Medicine, School of Medicine, University of Louisville, Louisville, KY USA; 7 Department of Cardiology, Paulista School of Medicine, Federal University of Sao Paulo, Sao Paulo, Brazil; 8 Department of Surgery, University of Sao Paulo Medical School, Sao Paulo, Brazil; 9 Department of Epidemiology and Population Health, University of Louisville, Louisville, KY USA

**Keywords:** Arrhythmias, Risk factors, Ventricular tachycardia

## Abstract

E-cigarette use has surged, but the long-term health effects remain unknown. E-cigarette aerosols containing nicotine and acrolein, a combustion and e-cigarette byproduct, may impair cardiac electrophysiology through autonomic imbalance. Here we show in mouse electrocardiograms that acute inhalation of e-cigarette aerosols disturbs cardiac conduction, in part through parasympathetic modulation. We demonstrate that, similar to acrolein or combustible cigarette smoke, aerosols from e-cigarette solvents (vegetable glycerin and propylene glycol) induce bradycardia, bradyarrhythmias, and elevations in heart rate variability during inhalation exposure, with inverse post-exposure effects. These effects are slighter with tobacco- or menthol-flavored aerosols containing nicotine, and in female mice. Yet, menthol-flavored and PG aerosols also increase ventricular arrhythmias and augment early ventricular repolarization (J amplitude), while menthol uniquely alters atrial and atrioventricular conduction. Exposure to e-cigarette aerosols from vegetable glycerin and its byproduct, acrolein, diminish heart rate and early repolarization. The pro-arrhythmic effects of solvent aerosols on ventricular repolarization and heart rate variability depend partly on parasympathetic modulation, whereas ventricular arrhythmias positively associate with early repolarization dependent on the presence of nicotine. Our study indicates that chemical constituents of e-cigarettes could contribute to cardiac risk by provoking pro-arrhythmic changes and stimulating autonomic reflexes.

## Abstract (web summary)

The use of E-cigarettes has increased despite the unknown long-term effects. Here the authors show that e-cigarette aerosols alter cardiac conduction, repolarization, and autonomic regulation in mice, contingent on the chemical composition of e-liquids and partly through parasympathetic modulation.

## Introduction

The use of electronic cigarettes (e-cigs) has reached epidemic proportions amidst perceptions that it is safer than smoking. In the past 4 years, youth use of e-cigs has surged, with more than one quarter of high schoolers and one tenth of middle schoolers in the United States reporting current use before the COVID-19 pandemic^1^. Along with conflicting reports that e-cigs could perpetuate smoking or aid quitting^2–4^, the finding that e-cigs may increase smoking initiation has fueled intense debate on the merits and harms of e-cigs^5,6^. Because e-cigs deliver nicotine without combusting tobacco, e-cig aerosol contains far less carbon monoxide, tar, and carcinogenic nitrosamines than cigarette smoke^7^, leading some to assert that e-cigs are reduced-harm products^8,9^. Yet, e-cigs could cause cardiopulmonary toxicity via other compounds. Several studies have shown that e-cig aerosols contain aldehydes and particulate matter (PM) at levels comparable to or above conventional cigarettes, which increase with flavors such as menthol^10–12^. The toxicity of these constituents at concentrations found in e-cig aerosols is still under investigation. Nonetheless, cases of e-cig or vaping-associated lung injury (EVALI) have recently highlighted the potential lethality of individual constituents in e-liquids^13^.

Although evidence for the cardiopulmonary toxicity of chronic e-cig use is largely restricted to animal studies, investigations in both animals and humans indicate that e-cigs promote hypertension, autonomic imbalance, and vascular dysfunction via nicotine and/or flavors^14–18^. Recent studies also suggest that nicotine-containing e-cig aerosol exposures acutely delay ventricular repolarization in humans^19^ while chronically inducing systolic dysfunction and atherosclerosis in a mouse model of atherogenesis^20^. Nevertheless, the impact of e-cigs on cardiac function has scarcely been studied; even the roles of the most common e-cig constituents—the solvents propylene glycol (PG) and vegetable glycerin (VG)—remain relatively unexamined.

E-cigs aerosolize PG and VG into PM to deliver nicotine and/or flavors to the lungs. Upon thermal degradation, PG and VG differentially produce the aldehydes acrolein, formaldehyde, and acetaldehyde^10^, which are estimated to account for 92% of the cardiovascular toxicity of cigarette smoke^21^. Inhalation of other types of PM at levels far lower than in e-cigs induces cardiovascular dysfunction^22–24^, and exposure to gaseous aldehydes at relevant concentrations induces adverse cardiovascular effects^25–30^. Hence, it is of high public health significance to evaluate the potential toxic effects of e-cig aerosols and to identify e-cig constituents contributing to these effects.

Given the widely reported link between smoking and sudden cardiac death, and the arrhythmogenic effects of nicotine, we evaluated the real-time effects of e-cig aerosols on cardiac electrophysiology upon acute inhalation. For reference, we also examined the effects of mainstream cigarette smoke (MCS), and to examine its contribution to e-cig toxicity, we studied the effects of acrolein, the aldehyde most implicated in the cardiovascular toxicity of smoking^21,31^.

In this work, we show that e-cig aerosols acutely induce arrhythmia, impair ventricular repolarization, and alter heart rate, supraventricular conduction, and HRV, consistent with autonomic imbalance. The findings of this study provide new insight into the cardiac risks of e-cigs relative to combustible cigarettes while identifying constituents and mechanisms that mediate these effects.

## Results

### E-cig exposures

In exposures with male mice, e-liquid consumption was greatest for PG:VG, followed in order by PG, E-menthol, VG, and E-Tobacco (Supplementary Table 3). In exposures with female mice, e-liquid consumption was 29% lower for PG:VG and 12% higher for E-Menthol relative to male exposures (Supplementary Table 3). TSP was also optically estimated to validate reproducibility and timing of exposures (Supplementary Table 3).

### E-cigs alter heart rate and HRV

**Fig. 1:**
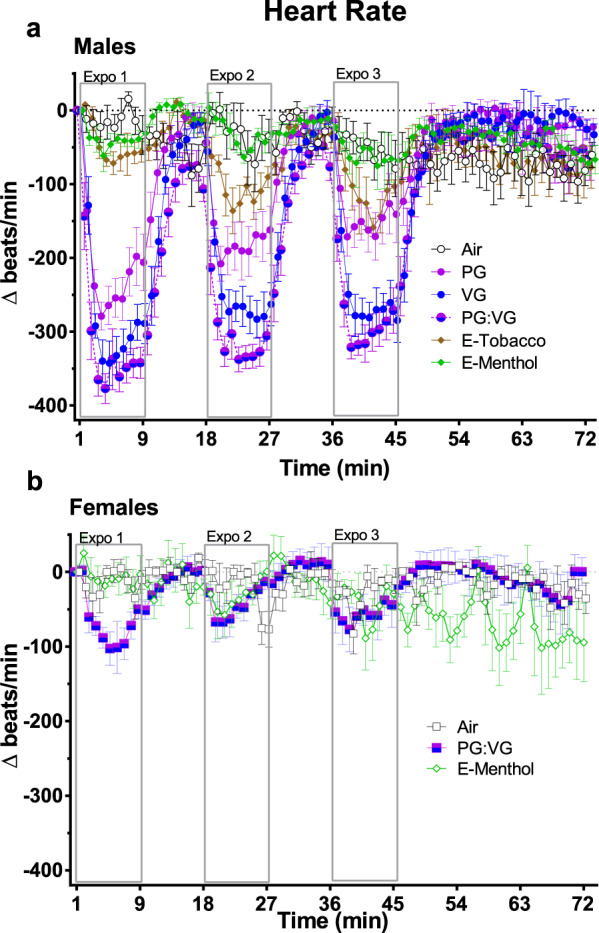
Timecourse of E-cig-induced alterations in heart rate by sex. Values represent 1-min simple means (±SEM) of change from 5-min baseline in heart rate during three 9-min inhalation exposures to e-cig solvents (PG, VG, and PG:VG, *n* = 8 male mice/exposure) or commercial e-liquids (E-Tobacco and E-Menthol, 2.4% freebase nicotine, *n* = 4 and *n* = 7 male mice, **a**, respectively). For all female exposures (**b**), *n* = 4 mice. Tall open boxes denote 9-min puff sessions (expo). Source data are provided as a Source Data file.

**Fig. 2:**
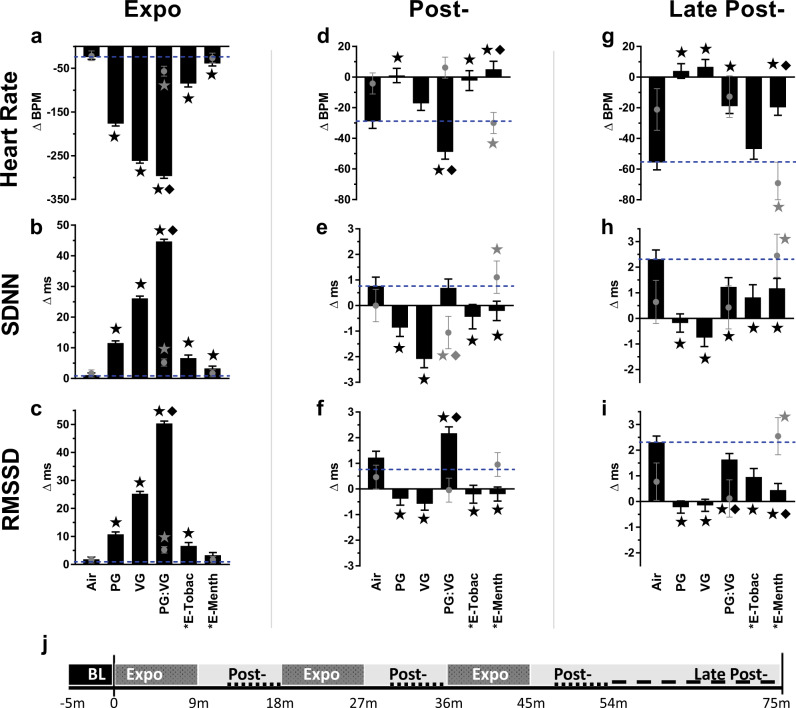
Inhalation exposure to E-cig aerosols alters heart rate and heart rate variability (HRV). Bars (males) or gray circles (females) represent least square means (±SEM) of change from 5-min baseline in heart rate and HRV (SDNN and RMSSD) at different exposure phases (**a**–**i**), and indicated by study timeline (**j**) with Expo denoting puff sessions, Post- denoting 4–9 min after each puff session, and Late Post- indicating 9–28 min after the final puff session. Dashed lines mark Air mean. Significance determined by two-sided *P* < 0.05 (vs. Air: star; between sexes: diamond) in mixed model analyses. Asterisk indicates nicotine present. “E-Tobac” and “E-Menth” denote E-Tobacco (*n* = 4) and E-Menthol (*n* = 7) among males. For Air, PG, VG, and PG:VG, *n* = 8 among males. For females, *n* = 4 per treatment. For averages of individual subjects by phase, and simple means and standard errors, see Supplementary Fig. 5. Source data, including all *p* values, are provided as a Source Data file.

In male mice, during exposure to e-cig solvent aerosols there was a dramatic decrease in HR accompanied by an increase in time-domain indices of HRV, including SDNN and RMSSD (Figs. 1 and 2 and Supplementary Fig. 3). Aerosols derived solely from PG:VG caused the greatest changes in HR and HRV when compared with air-only exposure (Air). Flavored nicotine-containing aerosols induced similar, but markedly smaller, HR and HRV effects than those solely from PG, VG, or PG:VG. In separate analyses of 10-s HR means among a representative subset of animals (*n* = 4) during the first puff session, exposure to PG:VG aerosols led to peak bradycardia by the fifth puff, which remained consistent until the end of the puff session and rapidly recovered during the first 4 min after exposure (Supplementary Fig. 4). Therefore, we separated our analysis of HRV and morphology parameters into 4 discrete phases—Baseline (BL), mid-exposure (Expo, all min of puff sessions), early after exposure (Post-, 4–9 min after each puff session), and late after exposure (Late Post-, 9–28 min after final puff session).

Male mice had significantly pronounced mid-exposure responses in HR and HRV relative to female mice (Fig. 1). In male mice all e-cig aerosols increased HR and decreased HRV relative to clean air (Fig. 2) during at least one post-exposure phase (Post- and/or Late Post-). Although early after exposure (Post-) SDNN did not differ from Air in PG:VG-exposed males, it was much lower in female mice relative to Air and male mice (both *P* < 0.05). Conversely, at Post-PG:VG, HR differed between sexes (*P* < 0.05) because it was unchanged in females (*P* > 0.05 vs. Air), and it remained unchanged during Late-Post in contrast to males. Females again contrasted with males after E-menthol, with persistent declines in HR (Post- and Late Post-, *P* < 0.05 vs. Air and Males), and elevations in SDNN (Post- and Late Post-, *P* < 0.05 vs. Air) and RMSSD (Late Post-, *P* < 0.05 vs. Air and Males).

### E-cigs alter supraventricular depolarization

**Fig. 3:**
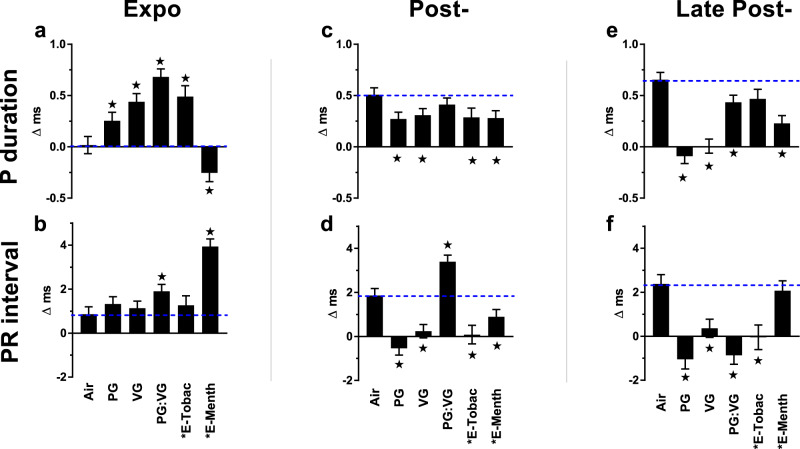
Inhalation exposure to E-cig aerosols alters supraventricular conduction. Bars represent least square means (±SEM) of change from 5-min baseline in P duration and PR interval according to exposure phases (**a**–**f**). Dashed lines mark Air mean. Significance determined by two-sided *P* < 0.05 (vs. Air: star) in mixed model analyses. Black asterisk indicates nicotine present. “E-Tobac” and “E-Menth” denote E-Tobacco and E-Menthol. Air (*n* = 7), PG (*n* = 6), VG (*n* = 7), PG:VG (*n* = 7), E-Tobac (*n* = 4), and E-Menth (*n* = 6). For averages of individual subjects by phase, and simple means and standard errors of each exposure, see Supplementary Fig. 6. Source data, including all *p* values, are provided as a Source Data file.

**Fig. 4:**
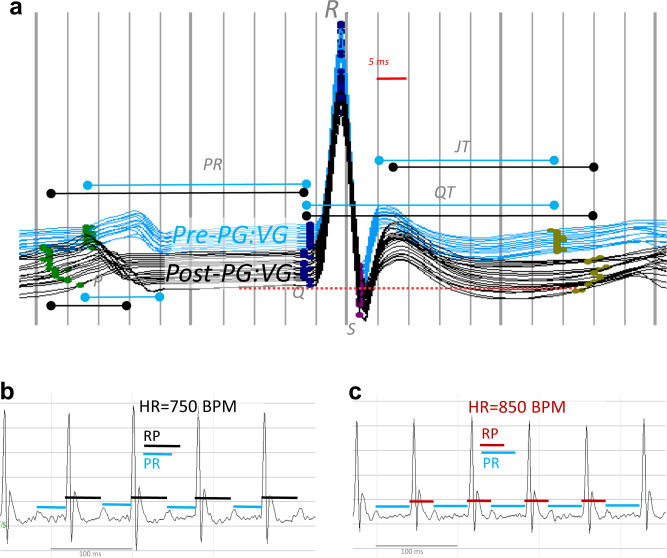
Representative ECG waveforms of E-cig exposed mice. **a** Waterfall plot of successive 1-min average ECG waveforms for a single mouse at pre-exposure (blue) and 4–9 min after (black) each PG:VG puff session. Horizontal lines indicate approximate mean P duration, PR, QT, and JT intervals for pre- (blue) and post-exposure (black). Waveform dots indicate P-begin (green), Q-begin (navy blue), R peak (navy blue), S (purple), and T-end (olive). After T-end, the next P appears as a rise above the isoelectric line (red dashed line for final 1-min average wave). **b** Normal conduction under physiological sinus rhythm with RP > PR in a single menthol e-cigarette-exposed mouse 30 s preceding onset of AV reentrant tachycardia (AVRT), as shown in **c**, when HR > 800 BPM and RP < PR, lasting 12 min.

Because males demonstrated greater chronotropic sensitivity to e-cig aerosols, we further analyzed their ECGs for changes in morphology. In males we saw P duration (Pdur) prolonged during exposure to all e-cig aerosols except E-menthol, which corresponded instead with shortened Pdur (Fig. 3). Exposures to E-Menthol and PG:VG were also associated with mid-exposure prolongation of the PR interval (Fig. 3 and Fig. 4a). Interestingly, with menthol aerosols, the two conflicting effects on supraventricular conduction corresponded with one male mouse developing atrioventricular reentrant tachycardia (AVRT) during exposure, characterized by a narrow QRS, HR > 800 BPM (mean = 838 BPM over 12 min), PR interval > RP interval, and RP interval > 9 ms, analogous to clinical guidelines^32^ (Fig. 4b, c). After exposure, all e-cig treatments corresponded with accelerated supraventricular conduction, generally characterized by shortened P and PR during Post- and/or Late Post- phases of exposure (Fig. 3).

### E-cigs affect ventricular repolarization

**Fig. 5:**
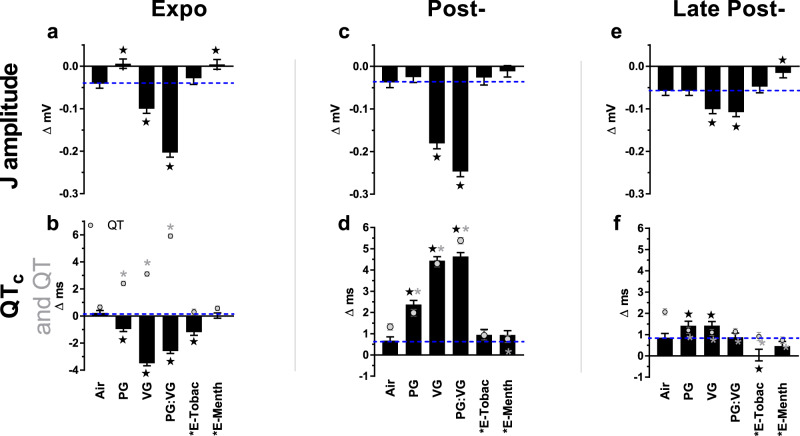
Inhalation exposure to E-cig aerosols alters ventricular repolarization. Bars represent least square means (±SEM) of change from 5-min baseline in J amplitude, QT_c_ interval, and uncorrected QT (gray circles) according to exposure phases (**a**–**f**). Significance determined by two-sided *P* < 0.05 (vs. Air: star for J amplitude and QTc and gray asterisk for QT) in mixed model analyses. Blue dashes mark Air mean. Black asterisk indicates nicotine present. “E-Tobac” and “E-Menth” denote E-Tobacco and E-Menthol. For averages of individual subjects by phase, and simple means and standard errors of Air (*n* = 8), PG (*n* = 7), VG (*n* = 7), PG:VG (*n* = 8), E-Tobac (*n* = 4), and E-Menth (*n* = 6) treatments, see Supplementary Fig. 6. Source data, including all *p* values, are provided as a Source Data file.

In male mice, all e-cig solvent exposures prolonged QT but shortened QT_c_ during the exposure phase (Fig. 5) concomitant with gross bradycardia and complementing our separate observation that murine QT only gradually adjusts to drastic shifts in HR, and thus QT_c_ initially overcorrects (Supplementary Fig. 2). In contrast, during the early post-exposure phase when HR fell within 30 BPM of Air, all e-cig solvent exposures prolonged both QT and QT_c_ relative to Air (Fig. 5). PG:VG and VG caused the greatest QT_c_ prolongation among all e-cig aerosols, amounting to 4.0-ms and 3.8-ms (+10%) vs. Air at post-exposure, respectively, and maintaining a slight prolongation at late post-exposure (0.6-ms each). QT_c_ was shortened during, and late after, exposure to E-Tobacco aerosols, but was unaffected by E-Menthol.

Nicotine-free e-cig aerosols also prolonged JT during exposure relative to Air, whereas most e-cig aerosols shortened JT at Post-, and all did at Late Post- (Supplementary Table 4). In all phases J amp (Fig. 5), a repolarization index modulated by I_to_ potassium current^33,34^, was decreased by nicotine-free aerosols from VG-containing solutions, whereas J amp was increased by E-Menthol during Expo and Late Post- and by PG-only during Expo. Finally, nicotine-bearing e-cig aerosols significantly depressed S amp—an index of ischemia—during exposure, whereas nicotine-free e-cig aerosols increased S amp (Supplementary Fig. 7).

### E-cigs evoke ventricular arrhythmia

**Fig. 6:**
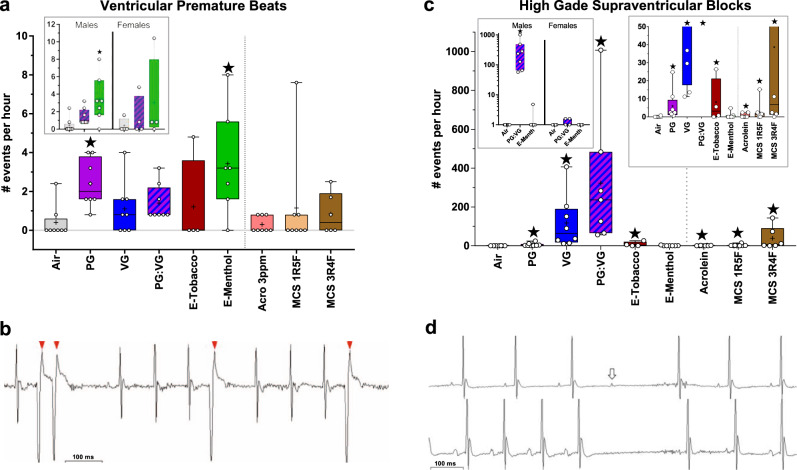
Inhalation exposure to E-cig aerosols increases cardiac arrhythmias. **a** Mean ± SEM number of arrhythmias per hour of ECG monitoring in males and both sexes (inset). **b** Representative VPBs (arrows) in an E-Menthol-exposed male. **c** Mean ± SEM number of high-grade SVB events per hour of ECG monitoring in males (smaller values in right inset) and females (left inset). **d** Representative high-grade SVBs, resembling atrioventricular block with non-conducted P waves (upper waveform, arrow), sinoatrial block lacking P waves (lower waveform); similar block events with noise preventing confirmation of P or its absence are not shown. Significance determined by two-sided *P* < 0.05 (vs. Air: star) in generalized estimating equations. For box and whisker plots, upper and lower box bounds indicate 25th and 75th percentile, with horizontal mid-line denoting median, “+” indicating mean, and circles indicating individual values (for males, *n* = 8 for Air, PG, PG:VG, VG, Acrolein [Acro], MCS 1R5F; *n* = 7 for E-Menth; *n* = 6 for MCS 3R4F; *n* = 4 for E-Tobacco; for females, *n* = 4/exposure). Source data, including all *p* values, are provided as a Source Data file.

In male mice, exposures to E-Menthol or PG aerosols increased ventricular premature beats (VPBs; Fig. 6a, b; mean ± SEM: 3.4 ± 1.0 events/h and 2.4 ± 0.4 events/h, respectively, both *P* < 0.05 vs. Air). To examine sex-related differences in arrhythmogenicity of e-cigs, we also analyzed females for arrhythmias. We found that exposure to E-Menthol in females corresponded with a similar, though not statistically significant, increase in VPBs (Fig. 6a). VPBs in males tended to increase during the exposure phases of E-menthol and E-Tobacco; however, e-cig aerosols of nicotine-free solvents did not recapitulate this trend (Supplementary Fig. 8). Exposures to all e-cig aerosols except E-Tobacco also corresponded with apparent elevations in VPB incidence proportions (percentage of treated animals with VPBs) vs. filtered air exposure (Supplementary Table 5).

We examined data from individual male mice for correlations between concurrent VPB counts and ECG changes, collectively across all e-cig treatments and exposure phases, or differentiating by exposure phases due to the time-varying effects on ECG parameters. Owing to the arrhythmogenic potential of nicotine^35–37^, we also analyzed data from nicotine-containing aerosols separately. We found that overall, VPBs correlated inversely with Pdur for all e-cig aerosols, and positively with PR for nicotine-containing e-cig aerosols (Supplementary Table 6). These data suggest that changes in supraventricular conduction may predict – or even underlie – the risk for e-cig-induced ventricular arrhythmia. During the exposure phase of nicotine-containing e-liquids, VPBs correlated inversely with HRV parameters, including LF, SDNN, RMSSD, and HF and positively with J amp (Supplementary Table 6). During the late post-exposure phases across all e-cigs, VPBs correlated inversely with HF, RMSSD, and PR, consistent with the arrhythmogenic effects of sympathetic dominance.

### E-cigs induce supraventricular arrhythmia

We identified extreme RR interval prolongation (RR > 300 ms), often consisting of non-conducted P waves, as “high-grade supraventricular block” (SVB) events (Fig. 6d). High-grade SVBs were markedly increased in males upon exposure to VG and PG:VG aerosols (Fig. 6c; mean ± SEM: 118 ± 48 events/h and 323 ± 127 events/h, *P* < 0.05 vs. Air), whereas PG-only and E-Tobacco aerosols were associated with slighter increases (8.2 ± 6.2 events/h and 6.0 ± 2.9 events/h, both *P* < 0.05 vs. Air). In contrast, E-Menthol exposure was not associated with changes in SVBs relative to Air. Nevertheless, all e-cig aerosols appeared to increase the incidence proportion of high-grade SVBs vs. filtered air (Supplementary Table 5). Interestingly, these events occurred primarily during exposure phases and rapidly subsided at post-exposure (Supplementary Fig. 8). Among females, exposure to PG:VG and E-menthol aerosols did not significantly affect high-grade SVB incidence (Fig. 6c).

Among males, frequency of high-grade SVBs correlated positively with changes in HRV (SDNN, RMSSD, HF, and LF) and inversely with changes in HR across all phases, particularly for e-cig exposures lacking nicotine (Supplementary Table 7). For ECG morphology, across all phases and particularly in the absence of nicotine, high-grade SVBs correlated positively with QT, JT, S amp, Pdur (a measure of atrial depolarization time), and PR, and inversely with J amp (Supplementary Table 7). When analyses were restricted to the mid-exposure phase, most correlations of high-grade SVBs with HRV and ECG morphology remained (except for S amp and PR). For e-cig exposures overall, high-grade SVBs correlated positively with Pdur (Supplementary Table 7). Because, in contrast, VPBs inversely correlated with Pdur overall (Supplementary Table 6), these findings together suggest that e-cigs may induce supraventricular and ventricular arrhythmias through opposing effects on a common target (i.e., atrial depolarization). Despite correlating positively with QT, high-grade SVBs correlated with QT_c_ inversely during the mid-exposure phase, consistent with QT_c_ initially overcorrecting via a lag in QT responding to gross heart rate changes (Supplementary Fig. 2). During the early post-exposure phase, when heart rate normalized, QT_c_ correlated positively with high-grade SVBs (Supplementary Table 7).

### E-cig solvents do not directly affect in vitro cardiomyocyte function

Neither PG nor VG (100 μM), either unheated or after heating at 200 °C or 700 °C, affected measures of cardiomyocyte beat rate, contractility, or viability and integrity (Supplementary Fig. 11).

### Acrolein and MCS exposures

Optical estimates of TSP validated reproducibility of MCS exposures and accorded with higher tar levels known for 3R4F relative to 1R5F (Supplementary Table 3).

### Acrolein and MCS alter heart rate and HRV

**Fig. 7:**
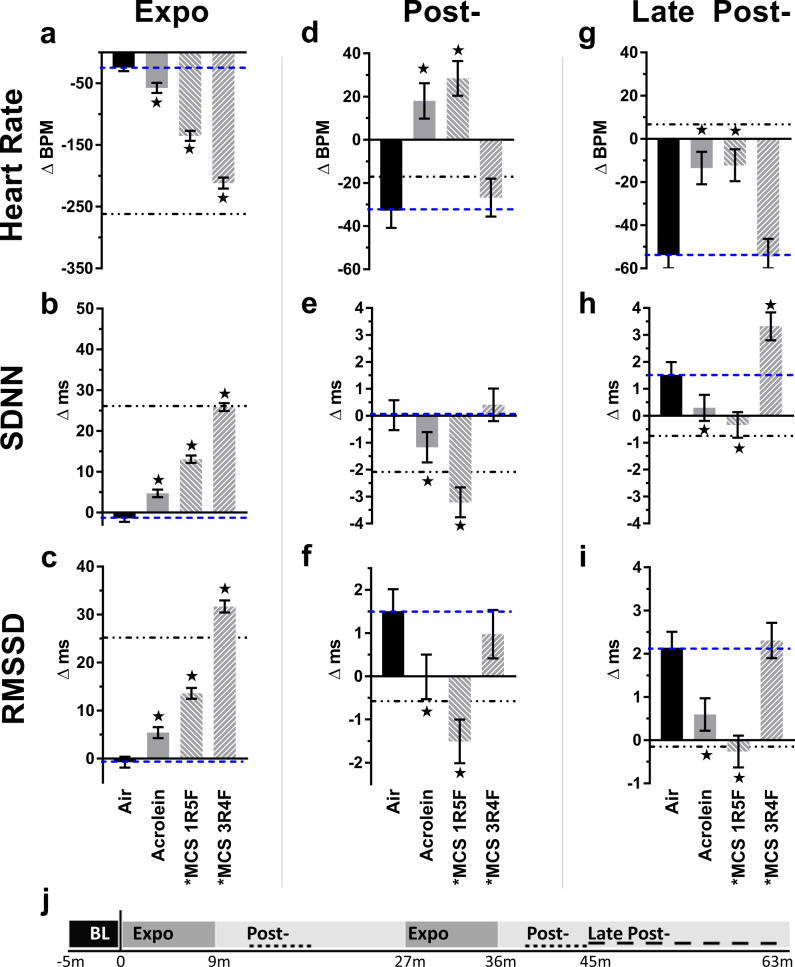
Inhalation exposure to MCS or acrolein alters heart rate and heart rate variability (HRV). Bars represent least square means (±SEM) of change from baseline in heart rate and HRV (SDNN and RMSSD) at different exposure phases (**a**–**i**), indicated by timeline below (**j**), with Expo denoting puff sessions, Post- denoting 4–9 min after each puff session, and Late Post- indicating 9–28 min after final puff session. Blue dashes mark Air mean (*n* = 8) and dot-dashes mark VG mean reference. Significance determined by two-sided *P* < 0.05 (vs. Air: star) in mixed model analyses. For Acrolein and MCS 1R5F, *n* = 8, and for MCS 3R4F, *n* = 6. Asterisk indicates nicotine present. For averages of individual subjects by phase, and simple means and standard errors, see Supplementary Fig. 9. Source data, including all *p* values, are provided as a Source Data file.

During exposure to acrolein and both types of MCS there was a decrease in HR and an increase in HRV, similar to e-cigs (Supplementary Fig. 4), with the greatest impact from 3R4F MCS, which in magnitude closely resembled the acute bradycardic and HRV-increasing effects of VG-only exposure (Fig. 7). In both post-exposure phases, mice exposed to either acrolein or 1R5F MCS showed elevated HR and depressed HRV relative to Air, resembling closely the effects observed with e-cig aerosols. In contrast, late after exposure full-flavor 3R4F MCS did not alter HR or RMSSD but elevated SDNN relative to Air. Notably, for MCS and acrolein, changes in HRV but not HR during exposure correlated with frequency of high-grade SVBs (Supplementary Table 7).

### Acrolein and MCS alter supraventricular depolarization

**Fig. 8:**
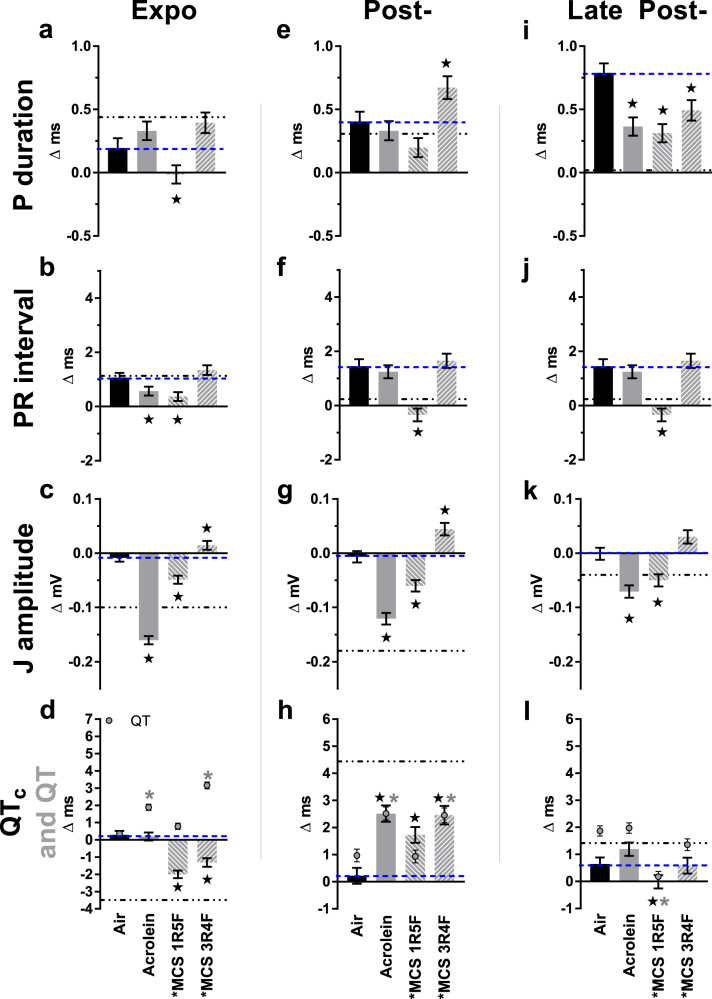
Inhalation exposure to cigarette smoke or acrolein alters supraventricular conduction and ventricular repolarization. Bars represent least square means (±SEM) of change from 5-min baseline in P duration, PR interval, J amplitude, QT_c_ interval, and uncorrected QT (gray circles) according to exposure phases (**a**–**l**). Blue dashes mark Air mean (*n* = 8) and dot-dashes mark VG mean as reference. Significance determined by two-sided *P* < 0.05 (vs. Air: star or, for QT, gray asterisk) in mixed model analyses. For Acrolein and MCS 1R5F, *n* = 8, and for MCS 3R4F, *n* = 6. Black asterisk indicates nicotine present. For averages of individual subjects by phase, and simple means and standard errors, see Supplementary Fig. 10. Source data, including all *p* values, are provided as a Source Data file.

Relative to Air, ultralight 1R5F MCS exposure corresponded with shortened Pdur and PR during exposure, in contrast with VG-only e-liquids (Fig. 8). Neither MCS nor acrolein exposure recapitulated the juxtaposing mid-exposure effects of E-Menthol between Pdur and PR. After exposure, 1R5F MCS corresponded with shortened PR (at Post- and Late Post-) and Pdur (at Late Post-) similar to most e-cig aerosols, whereas acrolein and 3R4F MCS only recapitulated these Pdur-shortening effects late after exposure.

### Acrolein and MCS alter ventricular repolarization

Exposure to acrolein or to either type of MCS recapitulated the effects of exposure to e-cig aerosols on the duration of ventricular repolarization during the Post-exposure phase, with QT_c_ prolonged by 6% after 3R4F, 6% after acrolein, and 4% after 1R5F (Fig. 8). Unlike e-cig aerosols, 3R4F MCS exposure corresponded with significantly prolonged JT during the Post-exposure phase (Supplementary Table 4) despite a HR comparable to Air. Conversely, as seen with e-cig aerosols, shortened JT occurred during the Late Post- phase following acrolein, 1R5F MCS, or 3R4F MCS (Supplementary Table 4). Similar to solvents containing VG (which thermally degrades to acrolein), acrolein and 1R5F MCS exposures led to a decrease in J amp relative to Air through all monitoring phases (Fig. 8). Conversely, exposure to 3R4F MCS (which has up to 10-fold the nicotine and tar of 1R5F MCS^38^) was associated with increased J amp during and shortly after exposures, resembling the mid- and late post-exposure effects of E-Menthol and PG-only. Like e-cig aerosols with nicotine, 3R4F MCS was associated with significantly depressed S amp during exposure, whereas acrolein concomitantly increased S amp similar to nicotine-free e-cig aerosols (Supplementary Fig. 7). Exposure to low-nicotine 1R5F had no effect.

### Acrolein and MCS evoke supraventricular block

In contrast with e-cig aerosols, neither acrolein nor MCS from either reference cigarette was associated with significantly altered frequency of VPBs relative to Air (Fig. 6a). VPBs across all phases correlated positively with acute changes in PR and J amp among all nicotine-containing aerosols including MCS and e-cigs (Supplementary Table 6), suggesting nicotine-bearing aerosols might trigger ventricular arrhythmias by uniquely altering atrioventricular conduction and/or early repolarization. Exposure to acrolein, 1R5F MCS, and 3R4F MCS, corresponded with significant increases in high-grade SVB events (0.9 ± 0.4, 2.7 ± 1.8, and 38.7 ± 23.9 events/hour, respectively, all *P* < 0.05 vs. Air), but did not induce as extreme of an increase as seen with nicotine-free e-cig solvents (Fig. 6c). MCS and acrolein exposures had positive correlations between high-grade SVB events and HRV changes during exposure (Supplementary Table 7). Upon acrolein inhalation, high-grade SVB events correlated inversely with QT_c_ during the exposure phase and inversely with JT in the early post-exposure phase. When collectively analyzed across all phases and exposures, the parameters most strongly correlated with high-grade SVB events were QT, SDNN, RMSSD, LF, and HF, which correlated positively, and HR, which correlated inversely (Supplementary Table 8).

### Role of nicotine in ventricular arrhythmogenesis

Due to the unique acute effects of nicotine-containing aerosols on repolarization, we tested whether nicotine modified the acute relationship between repolarization indices and VPB frequency. Interestingly, there was an inverse relationship between S amp and VPBs for nicotine-containing aerosols, such that a 0.01-mV depression in S amp (relative to baseline) associated with a 20% increase in VPBs, whereas S amp did not associate with VPBs for nicotine-free aerosols (Table 1 and Supplementary Fig. 12). Likewise, a 0.1-mV increase in J amp was associated with a 151% increase in VPBs for nicotine-containing aerosols, with no such associations for nicotine-free exposures. Notably, nicotine had near-significant interactions with the association between VPBs and both J and S amplitudes (Table 1), suggesting nicotine may modify the relationship between VPBs and early repolarization.

**Table 1:** Estimated association of changes in ECG repolarization parameters with VPB incidence upon inhalation exposure to nicotine-free and nicotine-containing aerosols

	Effect estimate (%)	95% CI	*P*-value	Interaction *P*-value
S amplitude (change in VPB incidence per 0.01-mV decrease from baseline)				
Nicotine-free exposures	−3.1	(−12.4, 7.1)	0.5362	0.0730
Nicotine aerosols	20.2^a^	(4.6, 38.2)	0.0095	
J amplitude (change in VPB incidence per 0.1-mV increase from baseline)				
Nicotine-free exposures	−15	(−48, 39)	0.5169	0.0909
Nicotine aerosols	151^a^	(46, 329)	0.0008	

^a^Significant association determined by generalized estimating equation and two-sided *P* < 0.05.

### Parasympathetic inhibition during PG:VG exposure

To test the role of the parasympathetic branch in the cardiac effects of e-cig aerosols, mice were pretreated with the muscarinic inhibitor atropine (0.1 mg/kg ip) or saline vehicle and exposed to PG:VG. Neither atropine nor saline injection significantly altered ECG or HRV at BL relative to animal-matched Air (all *P* > 0.10).

### Parasympathetic influence over E-cig effects on heart rate and HRV

**Fig. 9:**
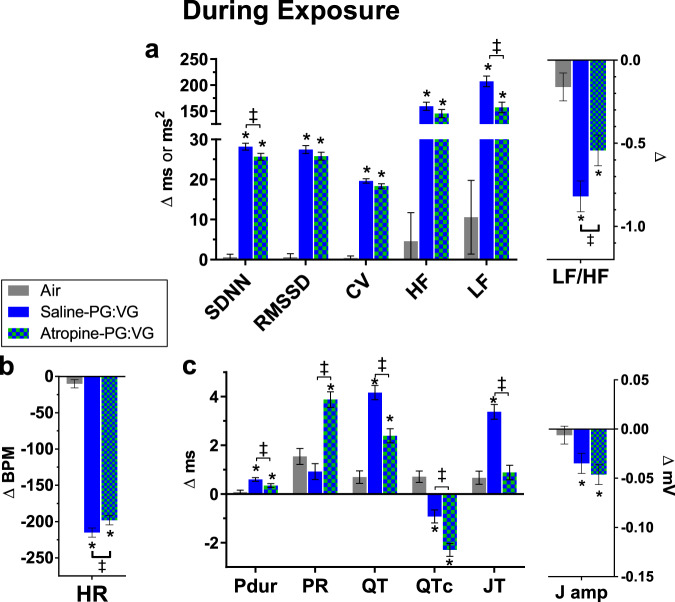
Muscarinic receptor blockade alters electrophysiologic and autonomic effects of E-cig aerosols during exposure. Bars represent least square means (±SEM) of change from 5-min baseline during exposure (*n* = 6/exposure) in HRV indices (**a**), heart rate (**b**), and ECG morphology (**c**). Significance determined by two-sided *P* < 0.05 (vs. Air: asterisk; atropine-PG:VG vs. Saline-PG:VG: two-barred cross) in mixed model analyses. Pdur indicates P duration and J amp indicates J amplitude. For averages of individual subjects by phase, and simple means and standard errors, see Supplementary Figs. 15–17. Source data, including all *p* values, are provided as a Source Data file.

Atropine modestly attenuated the PG:VG-associated bradycardia, increases in SDNN and LF, and decrease in LF/HF during exposure (Fig. 9). Early after exposure (Post-), there was no modification by atropine in the PG:VG-associated increase in HR and decrease in HRV (all *P* > 0.10 vs. Saline-PG:VG; Supplementary Fig. 13). Late after exposure (Late Post-, 9–27 min), atropine significantly diminished PG:VG-induced declines in HRV by 26–29% (*P* < 0.05 vs. saline-PG:VG; Supplementary Fig. 14), indicating pretreatment with a parasympathetic blocker attenuated sympathetic dominance after exposure.

### Parasympathetic mediation of E-cig impacts on depolarization and repolarization

Atropine attenuated Pdur prolongation while potentiating PR prolongation during PG:VG exposure relative to Air and Saline-PG:VG (Fig. 9); Atropine-PG:VG also corresponded with a greater PR prolongation than induced by PG:VG alone (mean difference ±SE, 1.0 ± 0.3 ms, *P* < 0.05). Likewise, atropine prevented PR shortening at early post-exposure (Supplementary Fig. 13) and continued to attenuate it during late post-exposure relative to Saline-PG:VG (Supplementary Fig. 14). After exposure, atropine did not modify PG:VG-induced Pdur shortening. Collectively, these data suggest parasympathetic responses may mitigate AV conduction delay during e-cig-induced bradycardia while facilitating AV conduction acceleration when heart rate increases thereafter.

During exposure, although atropine minimally attenuated PG:VG-induced bradycardia, it nearly halved PG:VG-induced QT prolongation and abolished PG:VG-induced JT prolongation (Fig. 9). Atropine also accentuated PG:VG-induced QT_c_ shortening during exposure, but this was likely secondary to a lag in QT accommodating to rate changes (e.g., Supplementary Fig. 2). Parasympathetic blockade by atropine also abolished JT prolongation early after PG:VG exposure, while it enhanced J-wave depression through both Post- and Late Post- phases (Supplementary Figs. 13 and 14). Thus, even though atropine did not modify PG:VG-induced QT_c_ prolongation at Post-, it attenuated QT and JT prolongation during and early after exposure, indicating that e-cig solvent aerosols impede ventricular repolarization in part through parasympathetic reflexes.

### Parasympathetic influence on E-cig-induced arrhythmia

Treatment with atropine did not affect the frequency of VPBs or high-grade SVBs in mice exposed to PG:VG aerosols. However, the incidence proportions of each arrhythmia type with PG:VG exposure appeared lower with atropine pre-treatment than with saline pre-treatment or no injection (Supplementary Table 5).

## Discussion

In this study we compared the acute cardiac effects of aerosols generated from e-cig solvents without nicotine with those elicited by exposure to mainstream cigarette smoke, acrolein, and aerosols generated by flavored e-cigs. We found that e-cig aerosols induced arrhythmia, impaired ventricular repolarization, and altered several processes subject to autonomic modulation, including heart rate, supraventricular conduction, and HRV, consistent with autonomic imbalance. In both male and female mice, exposure to e-cig aerosols from either a nicotine-containing menthol solution or nicotine-free PG-alone increased the frequency of ventricular tachyarrhythmias (i.e., VPBs). However, VPBs were unaffected by VG-alone, tobacco-flavored e-liquid, acrolein, and combustible cigarette smoke. Aerosols from menthol-containing e-cigs elicited the greatest increase in VPBs accompanied by robust changes in atrial and AV conduction. In contrast, VG-only aerosols induced the most profound bradycardia, bradyarrhythmias (high-grade SVBs), and repolarization changes. Collectively, these results suggest that e-cig use could increase CVD risk by inducing autonomic imbalance and cardiac arrhythmias and that the magnitude and profile of responses may depend on chemicals in the e-liquids, such as nicotine, solvents, and flavors. Our findings also suggest electrophysiologic changes induced by e-cigs can be attributed in part to autonomic imbalance leading to muscarinic receptor-mediated proarrhythmic changes associated with ventricular arrhythmias, including accelerated AV conduction, prolonged ventricular repolarization, and decreased HRV. Notably, we found that e-cig solvent-induced bradyarrhythmias, bradycardia, and HRV changes were more pronounced in male than in female mice, suggesting that males may be more sensitive to the immediate cardiac impacts of e-cig solvents. Male mice are also more susceptible to bradypnea, cardiac depression, and mortality upon acute exposures to high levels of acrolein (100–275 ppm)^39^.

The ECG data presented here indicate that menthol-flavored e-liquids display unique cardiotoxicity. Among the e-cig aerosols tested, only those containing menthol accelerated atrial conduction (P) despite prolonging AV conduction (PR). In agreement with the presumed arrhythmogenicity of juxtaposing changes in atrial and AV conduction—exposure to menthol-containing aerosols was associated with increased VPBs in males overall (with comparable values in females), with a 12-min AVRT episode in one male. In contrast, tobacco-flavored e-liquid and combustible cigarette smoke exposures neither increased tachyarrhythmias nor paradoxically altered supraventricular conduction velocities. We posit that the high toxicity of menthol may be due to either its pharmacologic effects or its propensity to generate higher levels of aldehydes. Work in our laboratory has shown previously that e-cigs containing menthol generate acrolein and formaldehyde at levels higher than tobacco-flavored e-cigs, and induce acrolein metabolite in exposed mice at levels that exceed tobacco flavor and compare closely to mice exposed to cigarette smoke^10,12^. It has also been reported that menthol-containing e-cigs generate 2 to 4 times higher levels of benzene and toluene (respectively 4×- and 2×-greater^40^). However, because cigarette smoke, which contains 200 times more benzene and 10 times more toluene^7,41^, did not increase VPBs, the proarrhythmic effects of menthol-containing e-cigs do not seem related to benzene or toluene. Instead, given that e-cigs can produce acrolein^11^ and formaldehyde^10^ at levels observed in cigarette smoke, it seems likely that these aldehydes may partly mediate the arrhythmogenic effects of menthol-containing e-cigs. Indeed, the arrhythmogenic effects of acrolein^27,42^ or formaldehyde^43^ alone support this possibility.

In addition to generating high levels of VOCs, menthol-containing e-cigarettes can also affect the heart via specific cardiopulmonary and autonomic mechanisms. Prior observations suggest menthol augments sympathetic regulation in smokers^44^. Menthol stimulates the transient receptor potential M8 ion channel (TRPM8) to inhibit tracheal parasympathetic modulation^45^, reduce irritation, and diminish respiratory braking in mice during MCS exposure^46^. Further, TRPM8 governs cold reflexes that enhance sympathetic tone, which promotes P shortening^47^; concordant with our findings reported here with mice exposed to menthol-containing e-cig aerosols. Thus, menthol could both augment inhaled dose of e-cig aerosols^12^ and trigger VPBs, while simultaneously inhibiting SVBs, via autonomic reflexes and attendant changes in cardiac conduction. This is consistent with the inverse association of VPBs with both Pdur and HRV. These findings have high public health relevance particularly because 57% of e-cig-using U.S. high schoolers have been recently reported to vape menthol/mint-flavored e-liquids^1^, and this proportion might increase with bans on other e-liquid flavors.

Our studies showing that exposure to e-cig aerosols can increase arrhythmia provide insight into the health implications of e-cig use. Results of recent studies suggest that e-cigs may increase cardiovascular risk, inducing vascular dysfunction or increasing hypertension and sympathetic dominance^14–18,48,49^. The sympathetic effects of e-cigs have been attributed to nicotine^16^, which alone can induce arrhythmia when infused^35^ or inhaled^36^. However, there may also be significant contribution of other constituents as well. We saw a significant increase in VPBs with exposure to PG and a clear increase in VPB incidence proportion with PG:VG. Thus, PG may impose its own arrhythmogenic effects that are potentiated by menthol or additional flavorants in the menthol-containing e-liquid. These changes are particularly important because seemingly occasional VPBs (>0.5 per h) can predict cardiac mortality in humans^50^, and recurrent VPBs can independently induce cardiomyopathy^51^. Therefore, because acute exposures to e-cig aerosols containing PG or menthol induced >2 VPBs per h, it seems plausible that habitual use of e-cigarettes may increase the risk of adverse cardiac changes.

E-cig aerosols from VG lacking nicotine disproportionately induced bradyarrhythmias and bradycardia. As VG aerosols contain more acrolein than PG aerosols^10,11^, the differences in the cardiac effects of VG and PG may stem from acrolein, the inhalation of which promotes parasympathetic dominance and bradyarrhythmia^26,27,42^. Additionally, our data suggest these effects are inhibited by nicotine, which stimulates neuronal norepinephrine release. Nicotine exposure could also increase VPBs upon exposure to menthol-containing aerosols due to direct inhibition of Kv channels that modulate I_to_ repolarizing current and, in turn, the murine J amp^33,34^. We found that J amp, a measure of early repolarization, increased with exposures to nicotine-containing menthol aerosols and smoke from 3R4F cigarettes (which contain 5–7× the nicotine in 1R5F^11,38^) and positively correlated with VPBs upon exposure to nicotine-containing aerosols in general. Interestingly, nicotine-free PG aerosols had a similar effect on J amp and significantly increased VPBs, indicating that PG aerosols also affect both I_to_ and arrhythmia. Of note, several human studies have positively associated increases in J amplitude with fatal arrhythmia^52^.

Both acrolein and low-nicotine sources of acrolein, including VG e-liquids and 1R5F, caused J depression and did not increase VPBs. Because smoke from 1R5F and 3R4F cigarettes contain comparable levels of acrolein^11^, these data suggest that nicotine and acrolein may have opposite effects on ventricular repolarization (I_to_). Although aerosols from tobacco-flavored e-cigarettes neither altered J amp nor increased VPBs, our prior observations that they induce less striking increases in urinary metabolites of nicotine, acrolein, and formaldehyde than menthol-containing aerosols^12^ – and that they generate less formaldehyde and acrolein in aerosol^10,12^ – may account for these null observations. Although our observations implicate nicotine, acrolein, PG, and menthol in ventricular arrhythmogenesis, their individual effects are particularly challenging to disentangle, and further investigations are needed to delineate both the short- and long-term cardiac effects of these constituents as well as others (e.g., formaldehyde) both by themselves and in mixtures.

Although ventricular arrhythmias were significantly increased only by PG and menthol, most other e-cig aerosols, as well as acrolein and cigarette smoke, prolonged QT. QT prolongation predicts cardiac mortality and cardiac arrest^53^ so reliably that its occurrence has banished many drugs from the market and clinical trials^54^. Therefore, measurements of QT are critical to the evaluation of any new drug. Although some studies have linked smoking to QT_c_ shortening^55^, most indicate that smoking^56–60^ or e-cigarette use^19^ can prolong QT or related measures of ventricular repolarization. Our observations extend these findings, showing that exposure to aerosols containing e-cig solvents alone can also comparably prolong QT. Thus, the use of e-cigs could impair ventricular repolarization and increase risk for cardiac mortality. Moreover, the effects of e-cigs may differ from cigarettes in their duration and magnitude. Notably, the greatest QT prolongation was observed with PG:VG. Additionally, an initial difference between QT and QT_c_ during e-cig solvent exposures complemented our separate findings that murine QT gradually adjusts to—and thus QT_c_ initially overcorrects for—drastic shifts in HR (Supplementary Fig. 2).

Our data also suggest a potentially ischemic effect of nicotine. Acute ST depression is a clinical hallmark of myocardial ischemia that can stem from constriction and spasm of coronary arteries with either smoking or sympathetic activation^61–64^. Depression of the murine S wave may similarly indicate ischemia^33^. We found that nicotine-containing aerosols induce significant S depression, and these effects are positively correlated with VPB frequency. Conversely, nicotine-free e-liquids had minimal impacts on S amplitude, which did not fluctuate in parallel with VPBs. These data thus suggest that nicotine promotes ventricular arrhythmia through ischemic effects.

During exposure, nicotine-free e-cig aerosols induced marked bradycardia and HRV increases, far exceeding the effects of cigarette smoke, acrolein, or e-cigs with nicotine. Similarly, among all ECG changes during e-cig exposures, fluctuations in heart rate and HRV were most strongly associated with high-grade supraventricular block events, which were provoked disproportionately by nicotine-free e-cig aerosols and dissipated rapidly after exposure. The rapid dissipation of these effects is particularly noteworthy, as ECG and HRV assessments of the acute effects of e-cigs (±nicotine) in humans have involved sampling after presumably brief but unspecified intervals from exposure^16^. Recently, HRV and QT_c_ were found to decrease in volunteers during exposure to secondhand e-cig aerosols^65^. Yet, these exposures likely differed from our current ones in levels of nicotine, PM, and VOCs. Indeed, PM concentrations and concurrent HRV/ECG endpoints may bear a sinusoidal relationship, in which low-level exposures have an effect opposite to that seen at higher concentrations^22^. Nevertheless, the effects observed in human volunteers shortly after the use of e-cigs do align with our findings in mice shortly after exposures, including decreases in HRV and increases in HR. Notably, these effects were not nicotine-dependent (Figs. 1 and 2).

The arrhythmogenic effects of e-cig solvent aerosols likely stemmed from autonomic reflexes. Accordingly, direct exposure of human iPSC-CMs to either heated or unheated PG or VG negligibly altered contractile function, beat rate, or viability. Conversely, multiple cardiac effects of PG:VG inhalation exposure (prolonged repolarization during exposure, accelerated AV conduction, and sympathetic dominance after exposure) were independently associated with increased VPBs and attenuated by parasympathetic blockade. Additionally, we observed fewer mice had VPBs during PG:VG exposure with atropine pretreatment relative to saline or no pretreatment. Indeed, vagal stimulation can impair ventricular repolarization, and such effects may lead to ventricular tachyarrhythmia^66^. Nonetheless, the minimal attenuation by atropine of heart rate depression and HRV elevation during the PG:VG exposure phase suggest that e-cig solvents may alter chronotropy independent of parasympathetic activation (e.g., sympathetic withdrawal). These observations parallel others in which sympathetic blockade attenuated acrolein-induced increases in HRV but parasympathetic inhibition had no such effect^27^. Accordingly, stimulation of a purinergic receptor that mediates reflexes to inhaled cigarette smoke^67^ can induce acute bradycardia disproportionately via sympathetic withdrawal^68^. In contrast to its minimal impact during exposure, atropine robustly blunted PG:VG-induced reductions in HRV (SDNN, RMSSD, and HF) late after exposure, implicating parasympathetic withdrawal as an underlying cause of this sympathetic dominance. These data complement our prior observations that atropine can attenuate sympathetic dominance after inhalation exposures and that aerosol-induced autonomic imbalance can oscillate over time^69^.

Importantly, chronic repetition of the short-term impacts of e-cigs on heart rate and QT could eventually manifest as electrical remodeling, accompanied by QT prolongation and increased arrhythmia^70,71^. Moreover, the varied effects of e-cig constituents on QT may more robustly predict arrhythmia in susceptible individuals, such as those with Long QT Syndrome, who succumb to sudden cardiac death predominately in youth, prior to any diagnosis, and secondary to drugs that exacerbate underlying dysfunction. Thus, our findings increase the urgency of comprehensive assessments of the cardiotoxicity of e-cig constituents, and merit close epidemiologic scrutiny of the relationships between e-cigarette use and cardiac outcomes.

Despite its many strengths, our study has some limitations. First, signal noise occasionally prevented differentiation between AV block and sinoatrial (SA) block. Thus, the clinical relevance of high-grade SVBs in mice is unclear. Separately, phenotypic traits of rodents, such as obligate nasal breathing, heightened capacity for reflex bradycardia, and a diminished I_Kr_ current, might cause cardiopulmonary responses distinct from humans. Likewise, in contrast to naïve rodents, repeated exposure to e-cig aerosols may lead to tolerance in humans, thereby decreasing cardiopulmonary reflexes, especially in adults with prior smoking history. Thus, the data presented here may pertain most to the cardiac and autonomic effects of e-cigs in new users and youth, and potentially secondhand e-cig exposures. To this end, animals were allowed to recover for ≥3 days between treatments, and differences from air exposures were tested after normalizing parameters to the baseline immediately preceding each treatment, which for some parameters, but not all, differed by treatment number. We therefore could not exclude a potential influence of exposure acclimation throughout the protocol. Finally, even though e-cig solvents disrupted cardiac rhythm more overtly in males than females, a more comprehensive comparison of both sexes across the multitude of aerosols used may better resolve sex-dependent sensitivity to various e-cig constituents.

## Methods

### Animals

C57BL6/J mice were obtained from Jackson Laboratories (Bar Harbor, ME) and treated according to the Guide for the Care and Use of Laboratory Animals. All protocols were approved by the University of Louisville (UL) Institutional Animal Care and Use Committee (IACUC). Mice were housed under pathogen-free conditions in a UL vivarium under controlled temperature and humidity (mean ± SD: 72.0 ± 0.0 °F and 39.7 ± 14.6%) and 12 h:12 h light:dark cycle and provided standard chow diet (Rodent Diet 5010, 4.5% fat by weight, LabDiet; St. Louis, MO) and water ad libitum. Mice (*n* = 12) were anesthetized with 1.5% isoflurane, subcutaneously implanted with radiotransmitters (ETA-F10, DSI, Inc., St. Paul, MN) with ECG electrodes in a lead II position, and allowed at least 10 days for post-operative recovery.

### Exposures

Telemetered C57 male mice (*n* = 6–8 per treatment; 12–30 weeks old) were placed for 90 min per exposure in inhalation chambers with dividers to avoid signal crosstalk. On a given exposure day, mice were exposed to either HEPA- and charcoal-filtered air (Air), mainstream cigarette smoke (MCS; 3R4F or 1R5F, two 9-min puff sessions), or e-cig aerosol (blu, three 9-min puff sessions) with ≥3-day recovery between exposures (Supplementary Table 1). Female C57 mice (*n* = 4) were similarly implanted with telemeters and exposed to Air and a subset of aerosols (PG:VG and E-menthol). Tobacco product aerosols were delivered to animals in 5-L chambers at 3 L/min bias flow and generated using a cigarette-smoking robot with flexiWare6 (inExpose, SCIREQ, Montreal, CAN) according to International Standard of Organization protocols for cigarettes (2-s puff, 35 mL puff, 1 puff/min, 9 puffs/cigarette, 1 cigarette/session, 18 min washout between sessions, 2 sessions) and e-cig puff patterns (4-s puffs, 91 mL puff, 2 puffs/min, 18 puffs/session, 9 min washout between sessions, 3 sessions) as previously described^12^. All e-cig aerosols were generated using a bluPLUS + ™ battery (3.7 V) and puff patterns resembling normal human use topography^72,73^. E-cig solvents (100% PG, 100% VG, and equal ratio PG:VG at 50/50 v/v) were aerosolized from a refillable tank atomizer (Mistic bridge, 0.5 mL, 1.8 Ω; 7.6 W) and bluPLUS+ cartridges (2.4% freebase nicotine; Magnificent Menthol and Classic Tobacco) were aerosolized from prefilled cartridges (3.5 Ω; 3.9 W). PG:VG exposures were replicated in a cross-over design with pre-injection of atropine (0.1 mg/kg, *ip*; A0132, Sigma) or 100 µL saline vehicle 15 min before 5-min baseline to allow recovery from injection. Because 3R4F produces a high amount of CO and e-cigs produce low to undetectable levels of CO^74^, the 3R4F mainstream smoke was diluted into two exposure chambers while e-cig aerosol was delivered to only 1 exposure chamber. Total suspended particulate (TSP) matter was monitored real time with an in-line infrared forward scattering monitor (MicroDust Pro; Casella CEL Ltd., Bedford, UK). E-liquid consumption was measured by weighing refillable tank or bluPLUS+ cartridges before and after exposures (see ref. 12). Mice were exposed to acrolein using a custom system consisting of (1) certified permeation tube for acrolein volatilization (Kin-Tek; LaMarque, TX); (2) a ppbRAE Plus photoionization detector (PID; ppb Rae Industries) for monitoring acrolein levels real time (inline and upstream); and, (3) a customized exposure chamber (30 L; flow 7–9 L/min) with a cyclone distribution insert as described for a target concentration of 3 ppm^75^.

In separate experiments, human induced pluripotent stem cell derived cardiomyocytes (hiPSC-CMs) were also used to test the direct impacts of e-cig solvents, both heated and unheated, on cardiomyocyte contractile function, beat rate, and viability (see Supplement)^76^.

### ECG

Mice were continuously monitored in whole-body exposure chambers by ECG (DataART 4.01 DSI, Inc.) sampled at 1 kHz for 15 min of pre-exposure and 75 min following initiation of exposures. One-minute ECG parameter averages for each mouse were used to calculate change from the mean of the final 5 min of pre-exposure (baseline, Supplementary Table 2) and analyzed by treatment as described below (Statistics). Animals were returned to their home cages 75 min after exposure initiation. ECG morphology, HRV, and arrhythmia incidence were analyzed blind to treatment in ECGauto 3.5 (emka Technologies, Paris, FR), while removing signal artifacts. ECG morphology was assessed by automatically analyzing waveforms with a library of ≥50 manually-marked representative beats in accordance with prior procedures^77^, deriving intervals (PR, JT, QT, and a custom QT_c_), P duration, amplitudes (S, J, and mean from 15 to 0 ms before Q-begin serving as isoelectric line) as indicated in Supplementary Fig. 1. Our QT_c_ formula was analogous to the conventional murine formula^78^: QT÷√(RR÷100) but modified to QT÷∛(RR÷100) because we found it more resilient to stress-evoked acute changes in RR in separate mice (Supplementary Fig. 2). Waveforms with mean baseline QT < 30 ms or >50 ms were inspected and excluded from QT and JT estimates if T was routinely mismarked; only one mouse was excluded in this manner (on an MCS 3R4F exposure day). We used automated detection of QR complexes to analyze RR intervals for time-domain HRV (root mean square of successive differences [RMSSD], standard deviation of normal inter-beat intervals [SDNN], coefficient of variation [CV, = (SDNN/RR)*100] and power spectral HRV parameters (low frequency [LF] and high frequency [HF]) configured for mice as described^42,79^. Across all exposures, ECG parameter values were generated from the average of conduction cycles successfully analyzed within each 1-min period (mean ± SD success of 93.6 ± 2.7 % and 47.7 ± 6.3 % of beats for HRV and morphology, respectively). We also analyzed ECG recordings for arrhythmias, including ventricular premature beats (with couplets constituting 2 VPBs, and ventricular tachycardia ≥3 VPBS) and high-grade supraventricular block events (SVB; RR > 300 ms)^22,80^. One-min parameter means coinciding with ≥1 s of tachycardia were excluded from analyses of exposure effects on ECG morphology and HRV.

### In vitro cardiomyocyte treatments and physiology

Human induced pluripotent stem cell derived cardiomyocytes (hiPSC-CMs) were obtained and assayed by a commercial vendor, Axiogenesis (now NCardia; Cor.4U.; Ax-C-HCO2–4 M, lot CB458Cl_4M) in an in vitro screening platform as previously described^81^. With human iPSCs from a female donor, Axiogenesis used directed differentiation to generate cardiac myocytes through intermediate cardiac gene-specific antibiotic selection. Because these cells can be maintained for long periods in culture, beat spontaneously, and exhibit an electrophysiological profile similar to that of adult human cardiomyocytes^82^, we evaluated the impact of exposure to PG, VG, and their thermal degradation products over a period of 48 h using a plate-base electrical impedance contractility assay to monitor changes in beat rate, contractile amplitudes, and cell index (base impedance measurement that reflects changes in cell viability, membrane integrity, and cell attachment). These assays were performed on a previously established, deidentified, iPSC line by Axiogenesis, a commercial third party, and thus did not require UL IRB authorization as research on non-human subjects. To generate thermal degradation products, compounds were delivered to a drop-tube furnace consisting of a quartz tube configured in a vertical position and set to either 200 °C or 700 °C. Aerosols were then collected within a glass impinger and subsequently eluted in an ethanol solution (55% in PBS). Stock concentrations of thermal product solutions that were used for experiments were determined by molar equivalents of compounds initially added to the furnace prior to heating and collection.

### Statistics

All statistical analyses were conducted in SAS 9.4 (Cary, NC) with *P* < 0.05 considered significant. For ECG morphology and HRV, 1-min means were analyzed by exposure phase (baseline, mid-, post-, and late post-exposure) and session (1–3) due to opposing effects on heart rate depending on phase. Treatments were tested for differences from Air in change from baseline (Supplementary Table 2) using linear mixed models and repeated subjects, testing for a treatment*sex interaction where applicable and reporting least squared means and standard errors. Mixed models are particularly useful for minimizing the impact of missing values (e.g., signal noise) and accounting for correlations between time-points. Because e-cig had one more exposure session per treatment than MCS/acrolein, analyses were stratified by treatment category (e-cig or MCS/acrolein). Arrhythmia counts were analyzed by treatment phase, time-normalized to incidence rates (per h), and tested for treatment effects with generalized estimating equations with negative binomial distribution while matching by exposure session and phase. Because nicotine can directly alter conduction and induce arrhythmia, we further tested for interactions between nicotine (yes vs. no) and repolarization-related ECG parameters in predicting ventricular arrhythmia among male mice. For any interactions with *P* < 0.10, analyses were stratified by nicotine to examine the relationship between that parameter and arrhythmia. All reported differences and correlations are statistically significant (*P* < 0.05 vs. Air control or sex) unless otherwise stated.

## Supplementary information

Supplementary Information

## Data Availability

The data supporting the findings from this study are available within the manuscript and its supplementary information. Source data for all reported means/averages in box plots, bar charts, tables, dot plots, and line graphs are provided with this paper. Additional raw data can be obtained upon request through the American Heart Association Precision Medicine Platform (precision.heart.org) by contacting the corresponding author (alex.carll@louisville.edu) and following an agreement on data use. Source data are provided with this paper.

## Source data

Source Data

## Code availability

All code used for this study can be obtained upon request through the American Heart Association Precision Medicine Platform (precision.heart.org) by contacting the corresponding author and following an agreement on data use.
