# Supplementary material for: E-cigarettes and their lone constituents induce cardiac arrhythmia and conduction defects in mice
Source: Nat Commun. 2022 Oct 25;13:6088. doi: 10.1038/s41467-022-33203-1 (PMC9596490; doi:10.1038/s41467-022-33203-1)
Supplement: Supplementary file 1 — Supplementary Information [file 41467_2022_33203_MOESM1_ESM.pdf]

Supplemental Material

Supplementary Results

Exposure Baselines. At baseline just before E-Menthol, PG, and VG exposures, heart rate was higher than on the day of Air exposure ($P < 0.05$, *Supplementary Table 2*). Baseline JT was higher for PG:VG, and baseline J amp was higher for PG:VG, VG, Acrolein, and Saline-PG:VG relative to Air ($P < 0.05$, *Supplementary Table 2*). There were no other significant baseline differences from Air for any other aerosols or any other morphology parameters ($P > 0.05$, *Supplementary Table 2*).

Arrhythmia incidence proportions. Although we observed that acrolein, 1R5F MCS, and 3R4F MCS exposures had a higher incidence proportion of VPBs than Air, all e-cig aerosol exposures except E-Tobacco had even higher incidence proportions (*Supplementary Table 5*). We also observed a higher incidence proportion of high-grade SVBs with acrolein and MCS exposures when compared with Air (*Supplementary Table 5*).

Time course of Arrhythmias. In contrast to e-cigs, acrolein and MCS exposures did not appear to induce VPBs disproportionately during the exposure sessions (*Supplementary Fig. 8*).

Supplementary Tables.

	Males (n=8)	
**n=3**	**n=3**	**n=2**
Air	*3R4F MCS*	1R5F MCS
3R4F MCS	*Air*	PG:VG
E-Tobacco	E-Tobacco	Acrolein
1R5F MCS	1R5F MCS	Air
PG:VG	PG:VG	PG
Acrolein	Acrolein	VG
PG	PG	E-Menthol
VG	VG	
E-Menthol	E-Menthol	
**n=3** (one from each row above)		**n=3** (one from each row above)
Atro-PG:VG		*Sal-PG:VG*
Sal-PG:VG		*Atro-PG:VG*
	Females **n=4**	
	Air	
	PG:VG	
	E-Menthol	

Supplementary Table 1. Exposure Sequence. Animals were exposed with ≥ 3 days between exposures in descending order. Each column represents a unique exposure cohort. For each treatment, the corresponding atmosphere was generated and delivered in 3-5 separate experiments. Treatments in italics have corresponding cross-over.

	N	Rhythm	ECG Morphology					
	**N**	**HR** (BPM)	**QTc** (ms)	**JT** (ms)	**J amp** (mV)	**S amp** (µV)	**PR** (ms)	**Pdur** (ms)
Air	8	659±29	41.8±2.3	28.0±1.7	0.14±0.05	-465±204	34.9±1.8	11.7±1.0
ENDS								
E-Tobacco	4	696±24	40.4±1.4	27.3±1.5	0.13±0.04	-425±260	37.5±3.5	11.6±1.5
E-Menthol	7	739±31*	41.1±2.8	27.8±2.5	0.30±0.19	-401±207	37.7±5.8	11.1±0.9
PG	8	738±28*	42.2±2.4	30.5±2.4	0.36±0.17	-371±226	35.2±3.0	11.9±1.3
PG:VG	8	707±21	42.7±1.8	31.4±2.2*	0.45±0.21*	-376±203	36.0±4.4	11.5±1.1
VG	8	721±34*	42.7±2.5	31.1±2.5	0.43±0.25*	-379±206	36.9±5.3	11.7±1.3
MCS/Acrolein								
MCS 1R5F	8	700±34	42.7±2.9	30.6±3.2	0.27±0.10	-388±210	36.6±3.0	11.6±1.2
MCS 3R4F	6	682±46	41.1±1.8′	27.6±1.8′	0.11±0.05	-489±190	34.6±2.1	11.1±0.9
Acrolein	8	640±79	39.7±4.6	30.9±3.4	0.52±0.17*	-297±162	35.0±2.5	11.9±1.0
Inhibition Study								
Saline-PG:VG	6	711±27	42.6±2.0	28.9±1.9	0.40±0.19*	-360±220	38.5±9.1	11.6±1.4
Atropine-PG:VG	6	711±34	43.6±0.6	29.6±1.2	0.37±0.13	-361±195	35.5±2.7	11.5±0.8

Supplementary Table 2. Exposure Baselines. Baseline heart rate and ECG morphology 5-min averages (± standard deviation) immediately before initial exposure to corresponding aerosol. *P < 0.05 relative to animal-matched Air measured by linear mixed model with Dunnett post-hoc comparison. ′n=5 due to exclusion of one mouse with baseline QT < 30 ms due to T_{end} mismarking. Female mean (± standard deviation) baseline heart rates (n=4) were 716±32 for Air, 720±36 for PG:VG, and 703±38 for E-Menthol.

Males	**E-liquid consumed** (mg)		**Mean TSP per puff** (mg/m^3)	
PG	204.6	± 18.7	2.58	± 0.21
VG	126.8	± 28.4	4.51	± 0.20
PG:VG	286.7	± 7.3	5.15	± 0.06
E-Tobacco	90.5	± 17.0	5.57	± 0.16
E-Menthol	191.9	± 50.8	4.94	± 0.36
MCS 1R5F			1.60	-
MCS 3R4F			3.08	± 0.01
PG:VG atropine/saline	236.4	± 31.8	4.99	± 0.57
Females				
PG:VG	204.2	-		
E-Menthol	215.3	-		

Supplementary Table 3. Exposure Characteristics. Total suspended particulate (TSP) was estimated as mean 15-s concentration per puff using a MicroDust Pro (Casella) and 1-s measurements. Mass of e-liquid consumed was measured gravimetrically. Values indicate mean ± standard deviation. Single dash indicates no standard deviation possible due to a single measure. TSP values for females were unavailable. PG, VG, PG:VG, E-Tobacco (2.4% nicotine), and E-Menthol (2.4% nicotine) aerosols were generated by e-cigarette. In contrast, MCS was generated by combustible cigarette.

	PG	VG	PG:VG	E-Tobacco	E-Menthol	Acrolein	MCS 1R5F	MCS 3R4F	Saline + PG:VG	Atropine + PG:VG
**n**	7	8	7	4	6	8	8	6	6	6
**Exposure**	1.2 ± 0.2**	0.5 ± 0.2*	1.6 ± 0.2**	0.0 ± 0.2	0.0 ± 0.2	0.1 ± 0.2	-0.3 ± 0.2	2.4 ± 0.2**	2.7 ± 0.4**	0.2 ± 0.4
**Post-Expo**	-0.6 ± 0.2*	-0.9 ± 0.2**	-0.7 ± 0.2**	-0.4 ± 0.2	-0.6 ± 0.2*	-0.1 ± 0.3	-1.3 ± 0.3**	0.9 ± 0.3*	0.5 ± 0.2*	-0.1 ± 0.2
**Late Post-**	-1.1 ± 0.2**	-2.0 ± 0.27**	-2.1 ± 0.2**	-0.9 ± 0.2**	-1.3 ± 0.2**	-0.8 ± 0.2**	-2.3 ± 0.2**	-0.5 ± 0.2*	-1.4 ± 0.1**	-1.5 ± 0.1**

Supplementary Table 4. Effects of Exposures on JT interval. Values are expressed as mean difference (+/- SEM) from filtered air exposure (experimental time-matched) in change from baseline in ms. Significance determined by two-sided P (*: P < 0.05 and **: P < 0.001 relative to filtered air control) in mixed model analyses.

	Air	PG	VG	PG:VG	E-Tobacco	E-Menthol	Acrolein	MCS 1R5F	MCS 3R4F	PG:VG + Saline	PG:VG + Atropine
**<u>Males</u>**											
**VPBs**	25%	100%	63%	100%	25%	86%	38%	38%	50%	100%	67%
**High Grade SVBs**	13%	100%	100%	100%	75%	29%	38%	63%	83%	100%	83%
**<u>Females</u>**											
**VPBs**	25%			50%		75%					
**High Grade SVBs**	0%			50%		25%					

Supplementary Table 5. Incidence proportion of animals with arrhythmias during treatment.

**Treatment Type**	**Phase**	**Nicotine**		**HRV Spearman's *r***	**ECG morphology Spearman's *r***
E-cigs	All	Both			Pdur -0.13*
		No Nic.	*n.s.*		
		Nicotine		LF -0.27*	PR 0.29*
	Expo	Both	*n.s.*		
		No Nic.	*n.s.*		
		Nicotine		LF -0.52**, SDNN -0.40*, RMSSD -0.37*, HF -0.36*	J amp 0.36*
	Post	Both		*n.s.*	
		No Nic.			S amp -0.23*
		Nicotine		LF/HF -0.40*	PR 0.40*
	Late	Both		RMSSD -0.31*, HF -0.32*	PR -0.43**
		No Nic.		RMSSD -0.34[a], HF -0.36*	PR -0.53**
		Nicotine	*n.s.*		
MCS & Acrolein			*n.s.*		
All	All	Both			S amp -0.10[a]
		No Nic.	*n.s.*		
		Nicotine			PR 0.19*, J amp 0.16[a]
	Expo	Both	*n.s.*		
		No Nic.		HR -0.22**	QT 0.20*
		Nicotine		LF -0.28*	J amp 0.31*
	Post	Both			PR 0.16*
		No Nic.	*n.s.*		
		Nicotine		LF/HF -0.30*	PR 0.29*
	Late	Both		HR 0.24*, SDNN -0.23*, RMSSD -0.29*, HF -0.26*	PR -0.34**
		No Nic.			PR -0.44**
		Nicotine	*n.s.*		

Supplementary Table 6. Summary of significant correlations between ventricular premature beat (VPB) arrhythmias and ECG-derived parameters. *n.s.: n*o significant correlations ($P > 0.05$). Nicotine: nicotine-containing treatments only. No Nic.: nicotine-free treatments only. Both: all treatments included. Estimates derived from Spearman's rank-order correlation with significance determined by two-sided P (*: $P < 0.05$, **: $P < 0.01$, ***: $P < 0.0001$).

Treatment Type	Phase	Nicotine	HRV Spearman's r	Morphology Spearman's r
E-cigs	All	Both	HR -0.53***, SDNN 0.55***, RMSSD 0.59***, LF 0.63***, HF 0.60***, LF/HF -0.18**	QT 0.59***, J amp -0.35***, PR 0.19**, JT 0.17**, S amp 0.15**, Pdur 0.15*
		No Nic.	HR -0.56***, SDNN 0.60***, RMSSD 0.64***, LF 0.69***, HF 0.66***, LF/HF -0.18**	QT 0.66***, J amp -0.37***, PR 0.21**, JT 0.19**, S amp 0.14*
		Nicotine	*n.s.*	
	Expo	Both	HR -0.69***, SDNN 0.74***, RMSSD 0.74***, LF 0.72***, HF 0.69***	QT 0.61***, QTc -0.60***, J amp -0.42***, JT 0.19*, Pdur 0.20*
		No Nic.	HR -0.73***, SDNN 0.78***, RMSSD 0.79***, LF 0.77***, HF 0.72***	QT 0.66***, QTc -0.62***, J amp -0.46***
		Nicotine		Pdur 0.37*
	Post	Both	HR -0.49***, SDNN 0.54***, RMSSD 0.60***, LF 0.63***, HF 0.60***, LF/HF -0.25**	QT 0.62***, QTc 0.43***, J amp -0.38***, PR 0.26**, S amp 0.24**
		No Nic.	*n.s.*	
		Nicotine	LF/HF -0.35*	
	Late		*n.s.*	
MCS & Acrolein	All	Both	*n.s.*	
		No Nic.	*n.s.*	
		Nicotine	*n.s.*	
	Expo	Both	SDNN 0.35**, RMSSD 0.36**, LF 0.36**, HF 0.37**	
		No Nic.		QTc -0.38*
		Nicotine	*n.s.*	
	Post	Both	*n.s.*	
		No Nic.		JT -0.39*
		Nicotine	*n.s.*	
	Late		*n.s.*	

Supplementary Table 7. Summary of significant correlations between high-grade supraventricular block arrhythmias and ECG-derived parameters. Blank spaces indicate no significant correlations (P > 0.05). Nic.: nicotine-containing treatments only. No Nic.: nicotine-free treatments only. Both: all treatments, both nicotine-containing and nicotine-free, included. Estimates derived from Spearman's rank-order correlation with significance determined by two-sided P (*: P < 0.05, **: P < 0.01, ***: P < 0.0001).

**Treatment Type**	**Phase**	**Nicotine**	**HRV Spearman's *r***	**ECG morphology Spearman's *r***
All	All	Both	HR -0.42***, SDNN 0.43***, RMSSD 0.45***, LF 0.50***, HF 0.45***, LF/HF -0.13**	QT 0.46***, J amp -0.22***, PR 0.16**, Pdur 0.14**, JT 0.12*, S amp 0.10*
		No Nic.	HR -0.54***, SDNN 0.54***, RMSSD 0.58***, HF 0.58***, LF 0.62***, LF/HF -0.16**	QT 0.58***, J amp -0.29***, PR 0.19**, JT 0.15**, S amp 0.13*
		Nicotine		Pdur 0.20*
	Expo	Both	HR -0.62***, SDNN 0.64***, RMSSD 0.63***, LF 0.64***, HF 0.55***	QT 0.50***, QTc -0.50***, J amp -0.25**, Pdur 0.22**, JT 0.17*
		No Nic.	HR -0.74***, SDNN 0.77***, RMSSD 0.77***, LF 0.75***, HF 0.70***	QT 0.62***, QTc -0.64***, J amp -0.32**, JT 0.24**
		Nicotine		Pdur 0.31*
	Post	Both	*n.s.*	
		No Nic.	HR -0.42***, SDNN 0.46***, RMSSD 0.52***, LF 0.61***, HF 0.53***, LF/HF -0.20*	QT 0.60***, QTc 0.41***, J amp -0.34**, PR 0.24*, S amp 0.21*
		Nicotine	*n.s.*	
	Late		*n.s.*	

Supplementary Table 8. Summary of significant correlations between high-grade supraventricular block arrhythmias and ECG-derived parameters (ctd.). Blank spaces indicate no significant correlations (P > 0.05). Nicotine: nicotine-containing treatments only. No Nic.: nicotine-free treatments only. Both: all treatments, both nicotine-containing and nicotine-free, included. Estimates derived from Spearman's rank-order correlation with significance determined by two-sided P (*: P < 0.05, **: P < 0.01, ***: P < 0.0001

Supplementary Figures.

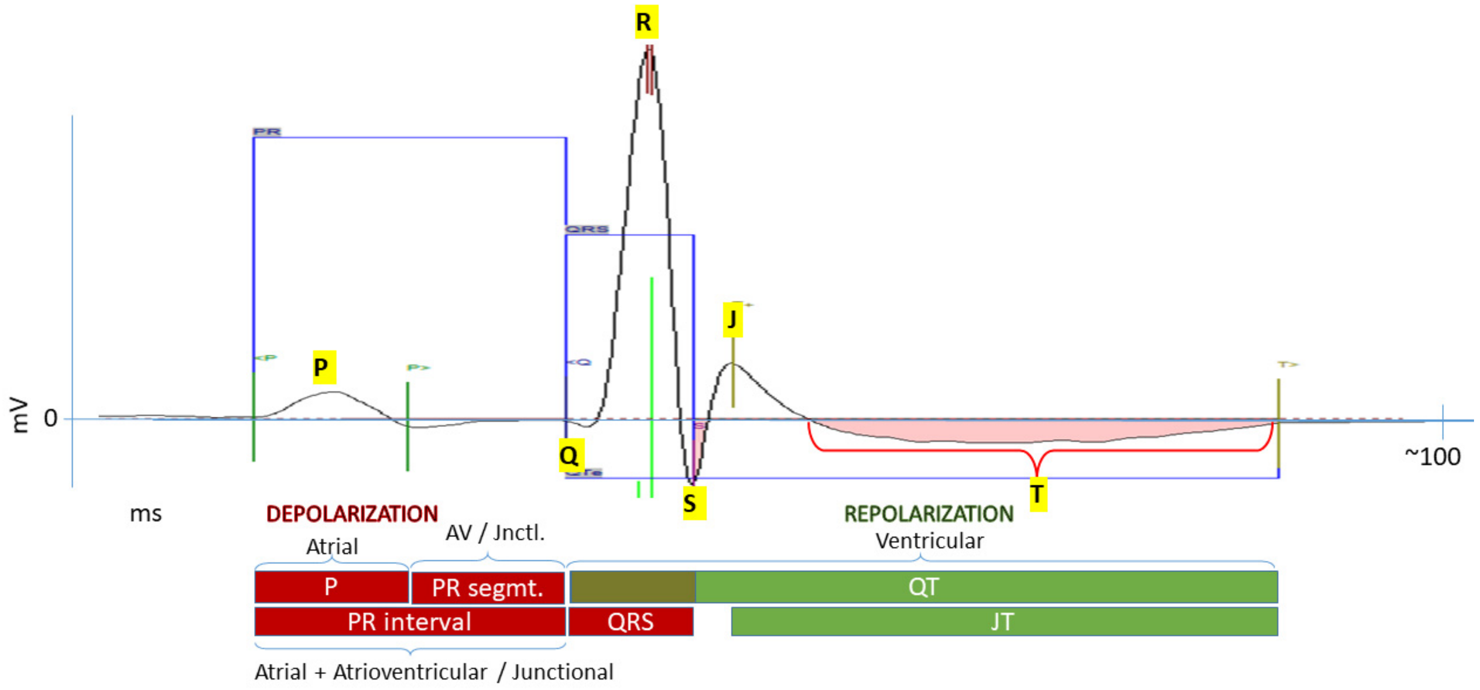

Supplementary Figure 1. Murine ECG landmarks and corresponding conduction periods in a single cardiac conduction cycle.

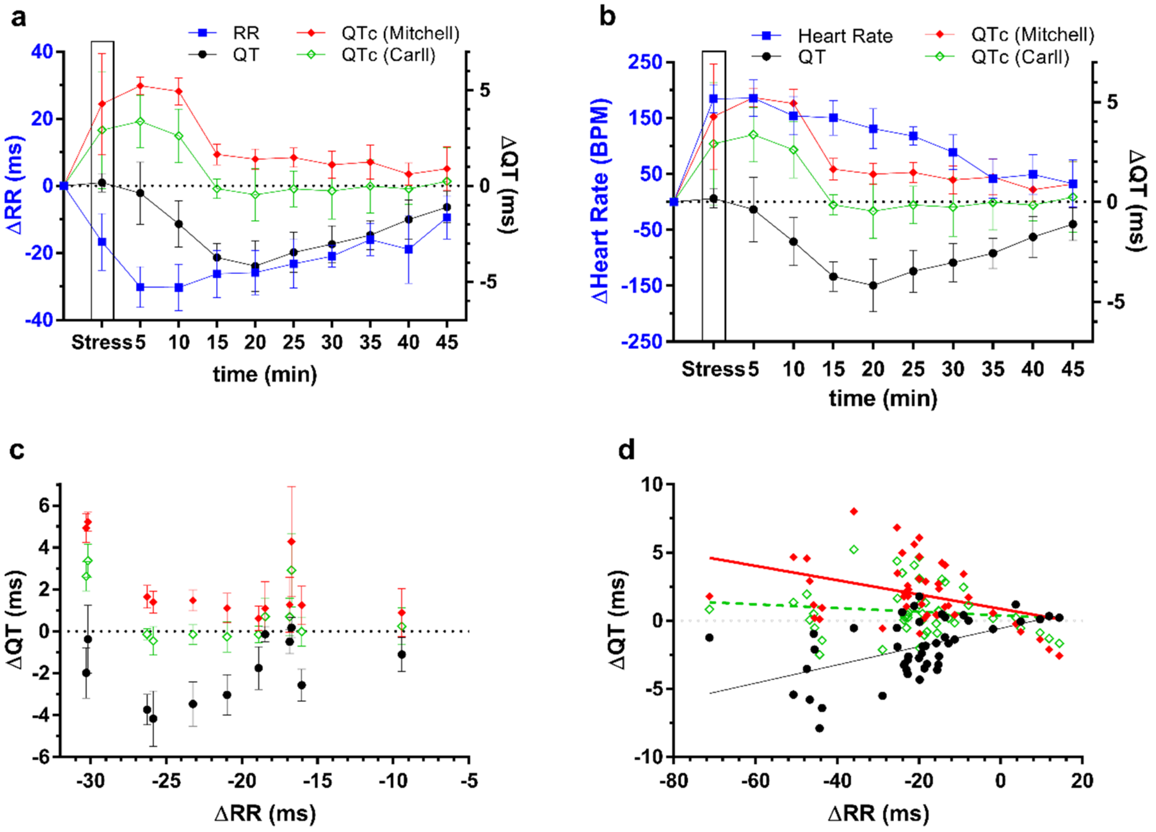

	QT	QT_c (Mitchell)	QT_c (Carll)
**Formula**		$\frac{QT}{\sqrt{(\frac{RR}{100})}}$	$\frac{QT}{\sqrt[3]{(\frac{RR}{100})}}$
r^2	0.28	0.15	0.02
**P-value**	0.0002	0.0079	0.4013

Supplementary Figure 2. QT-RR relationship in conscious telemetered mice during and after restraint stress. In a pilot study, male telemetered C57BL/6J mice (30 weeks, n=4) were challenged by placement in a restraining tube (CODA mouse tail cuff tubes, Kent Scientific) for five minutes in dorsal recumbency and then returned to home cages for recovery monitoring. **a** and **b,** 5-min mean differences (± SEM) from animal-matched baseline (average of 20 min in home cage immediately before stress) in RR (**a**, left y-axis), HR (**b**, left y-axis) or QT (right y-axis) either in its raw form (black) or corrected (QT_c) per the leading murine formula[1] (red) or a new murine formula (green). **c,** relationship between mean ΔRR and mean ΔQT (± SEM)—either uncorrected or corrected. **d,** linear regressions of individual animal ΔRR vs. either ΔQT or ΔQT_c. Table shows corresponding formulas, R^2, and P values from linear regressions, with significance determined by two-sided $P < 0.05$. RR and QT values were concurrently derived from full analyses of ECG morphology, whereas HR values were derived from RR interval-only analysis.

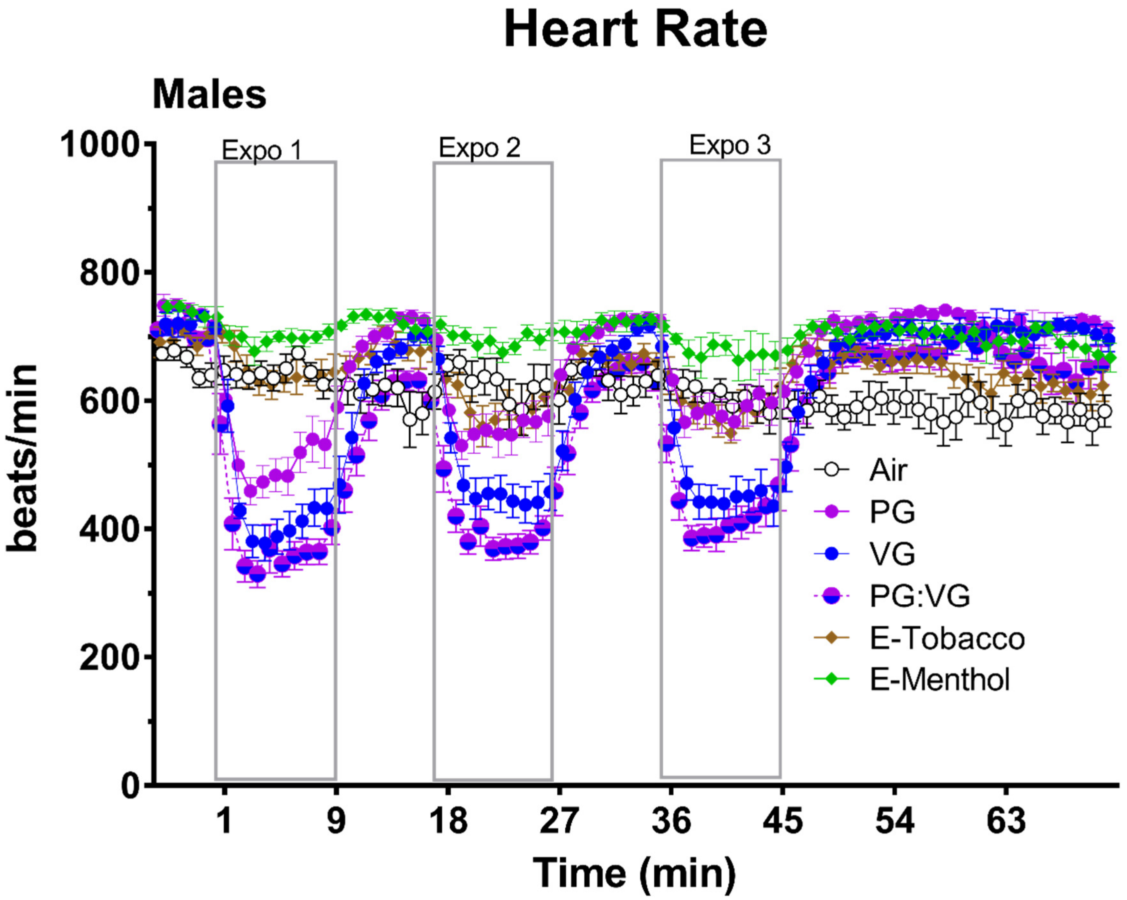

Supplementary Figure 3. Time course of E-cig-induced Alterations in Absolute Heart Rate. Values represent mean (± SEM) heart rate during three 9-min inhalation exposures to e-cig solvents (PG, VG, and PG:VG, n=8 males/exposure) or commercial e-liquids (E-Tobacco and E-Menthol, 2.4% freebase nicotine, n=4 and n=7 male mice, respectively). Tall open boxes denote 9-min puff sessions (Expo).

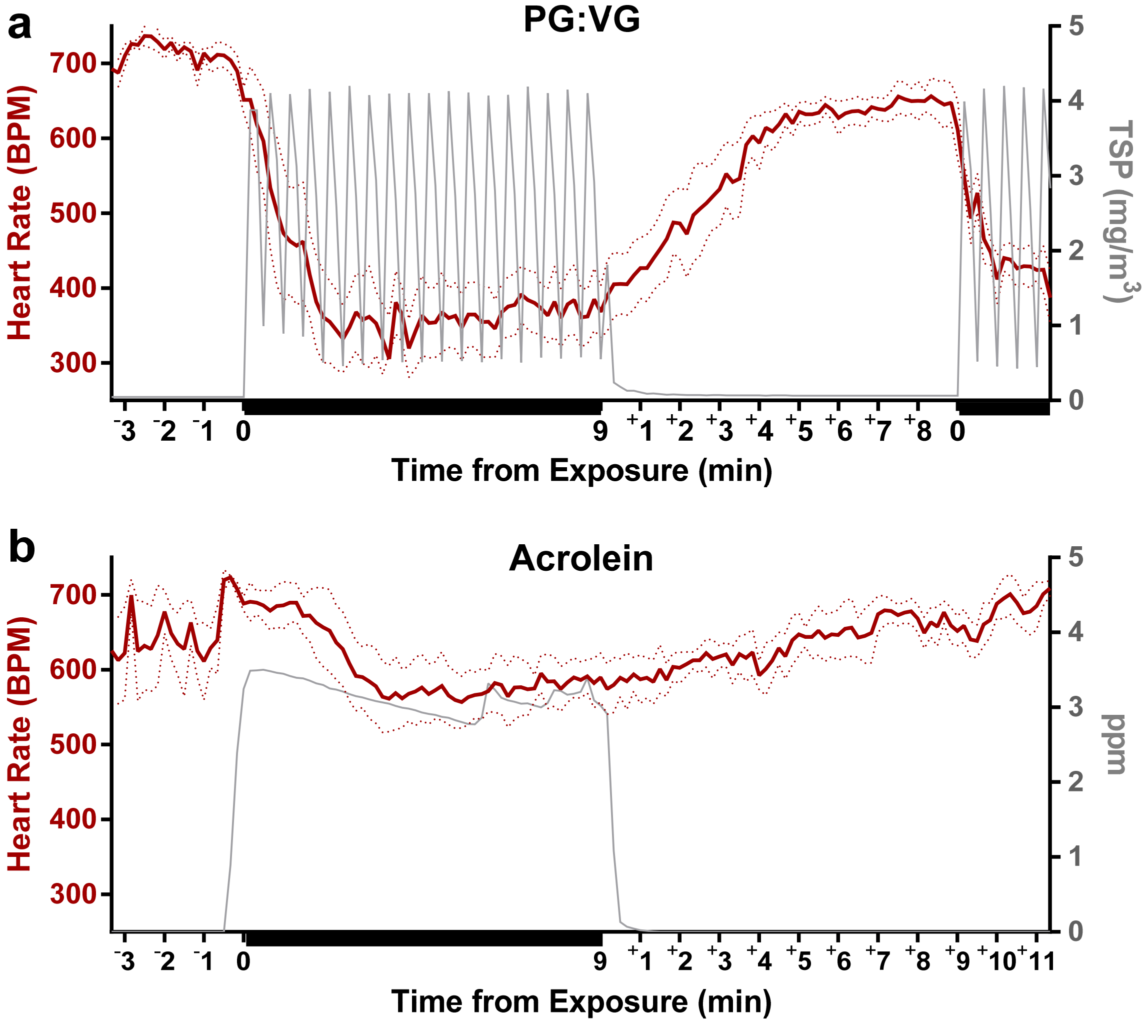

Supplementary Figure 4. Representative heart rate responses and concentrations of total suspended particulate (TSP) or acrolein gas in exposure chambers from a single experimental exposure session. Values are presented as 10-s averages of heart rate (± SEM; n=4 males each) or concentrations from a single test atmosphere generated during the initial exposure session to PG:VG (**a**) or acrolein (**b**). PG:VG puffs are indicated by spikes in TSP, in contrast to continuous 9-min acrolein exposure at a target 3 ppm concentration. Black bars indicate exposure sessions.

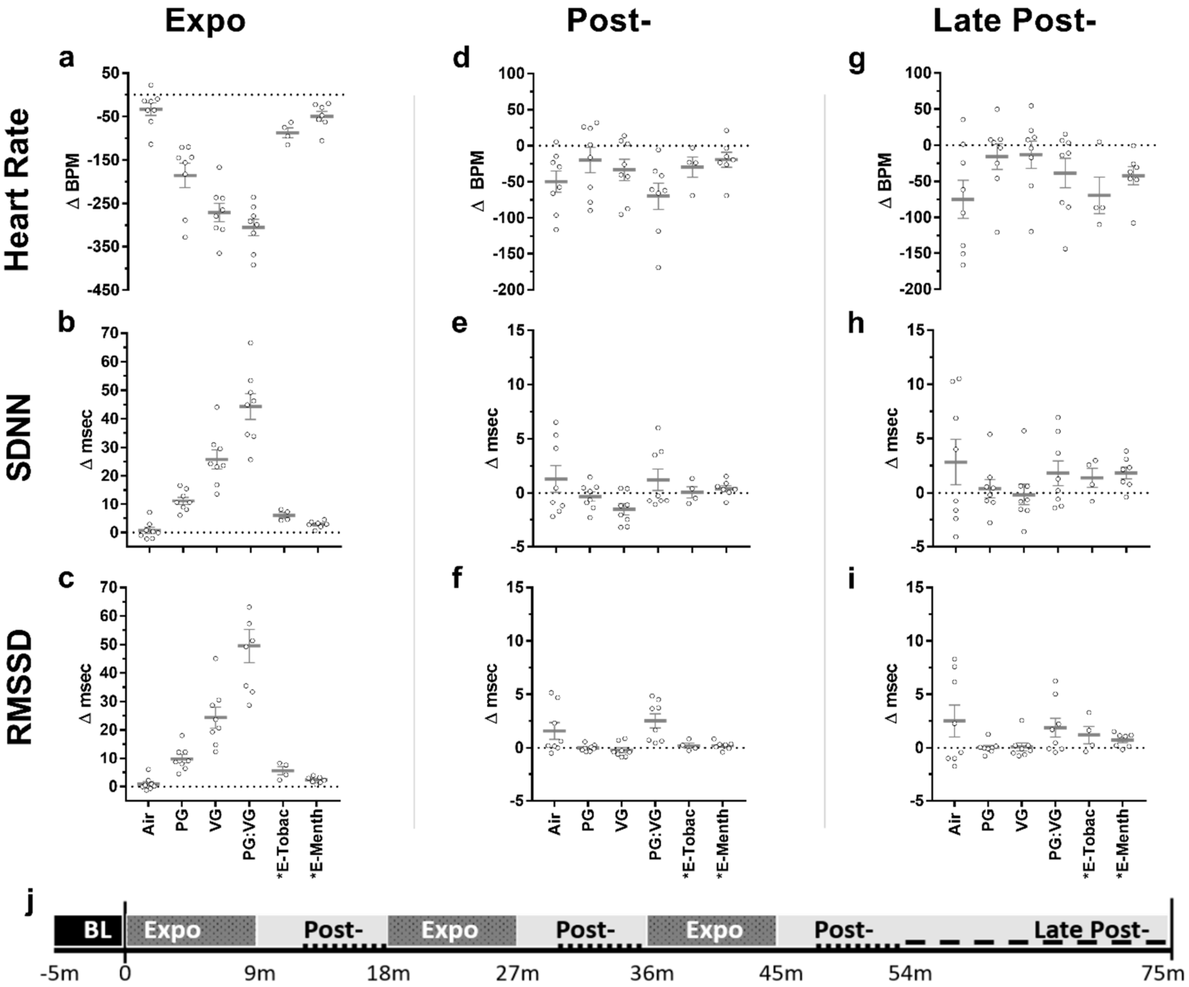

Supplementary Figure 5. Effects of E-cigarette Exposures on Heart Rate and HRV. Individual subject average changes from 5-min baseline in heart rate and HRV (SDNN and RMSSD, **a-i**), stratified by phase, with observed simple means and standard errors indicated by gray bar ± error bars. Phases are indicated by study timeline, **j**, with Expo denoting puff sessions, Post- denoting 4-9 min after each puff session, and Late Post- indicating 9-28 min after the final puff session. "E-Tobac" and "E-Menth" denote E-Tobacco (n=4) and E-Menthol (n=7). For Air, PG, VG, and PG:VG, n=8. For least square means and statistical comparisons, see **Fig. 2**.

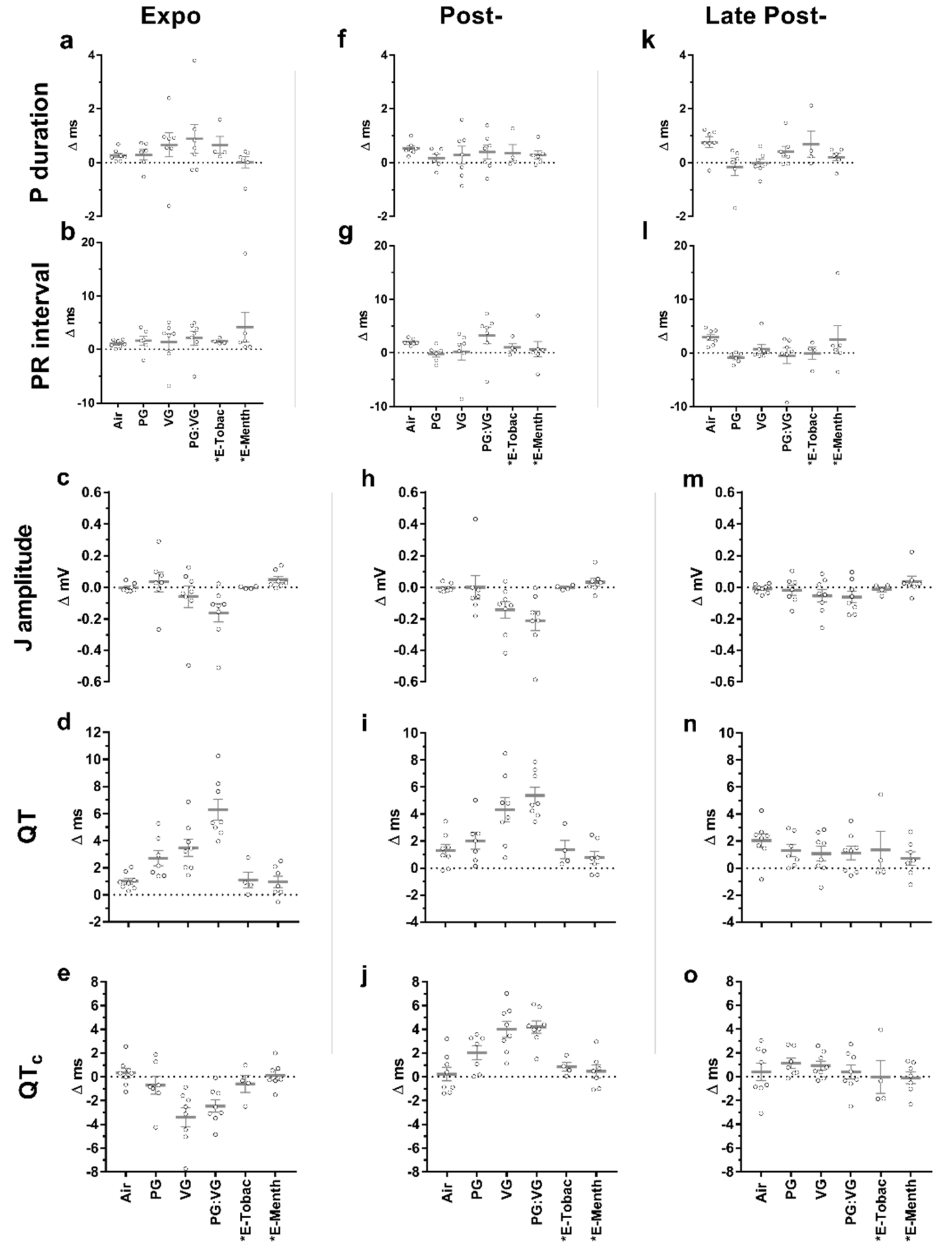

Supplementary Figure 6. Effects of E-cigarette Exposures on ECG Morphology. Individual subject average changes from 5-min baseline in ECG morphology parameters, stratified by phase, with observed simple means and standard errors indicated by gray bar ± error bars. Phases are indicated by study timeline below, with Expo denoting puff sessions, Post- denoting 4-9 min after each puff session, and Late Post- indicating 9-28 min after the final puff session. "E-Tobac" and "E-Menth" denote E-Tobacco and E-Menthol. For P dur and PR, Air n=7, PG n=6, VG n=7, PG:VG n=7, E-Tobac n=4, and E-Menth n=6. For J and QT, Air n=8, PG n=7, VG n=7, PG:VG n=8, E-Tobac n=4, and E-Menth n=7. For least square means and statistical comparisons, see **Fig. 3** and **Fig. 5**.

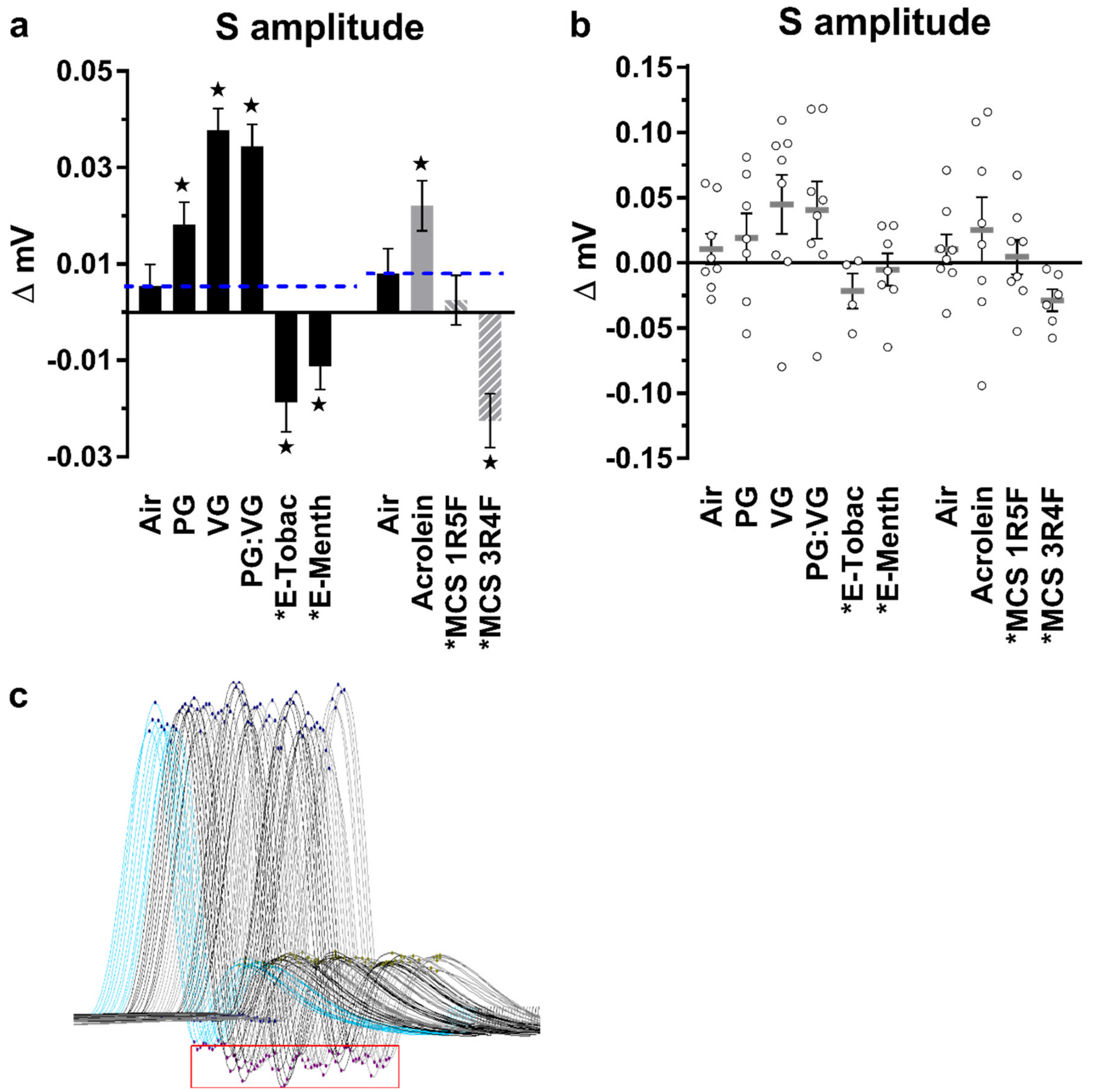

Supplementary Figure 7. Nicotine-containing e-cig aerosols induce S depression during exposure. a, bars represent least square means (± SEM) of change from 5-min baseline in S amplitude during exposure to E-cig aerosol, MCS, or acrolein. "E-Tobac" and "E-Menth" denote E-Tobacco (n=4) and E-Menthol (n=7). **b**, individual subject average changes from 5-min baseline in S amplitude with observed simple means and standard errors indicated by gray bar ± error bars. For Air n=8, PG n=7, VG n=8, PG:VG n=8, acrolein n=8, MCS 1R5F n=8, and MCS 3R4F n=6. **c,** representative 1-min average ECG waveforms generated from baseline (blue), three 9-min E-Tobacco puff sessions (black), or post-exposure (gray) in a single mouse. Significance determined by two-sided $P < 0.05$ (vs. Air, indicated by stars) in mixed model analyses. Asterisks indicate presence of nicotine.

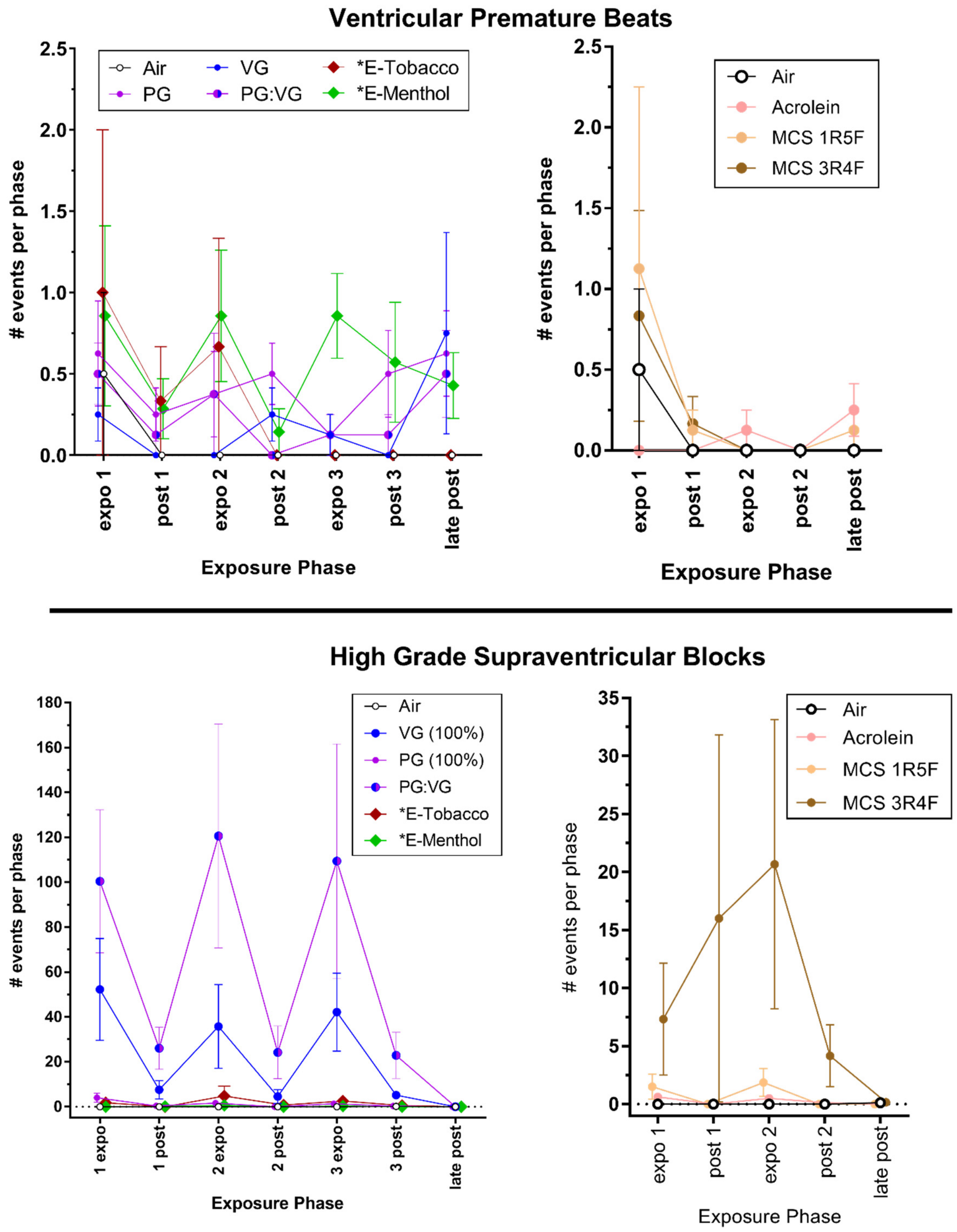

Supplementary Figure 8. Time course of Treatment-Associated Arrhythmias. Observed simple mean number of VPBs and high grade SVBs (± SEM) per phase in male mice. "Post" phases spanned 0-9 min post-exposure for e-cigs or 0-18 min post-exposure between acrolein and MCS sessions, and "Late Post-" phases spanned 9-27 min after final exposure session.

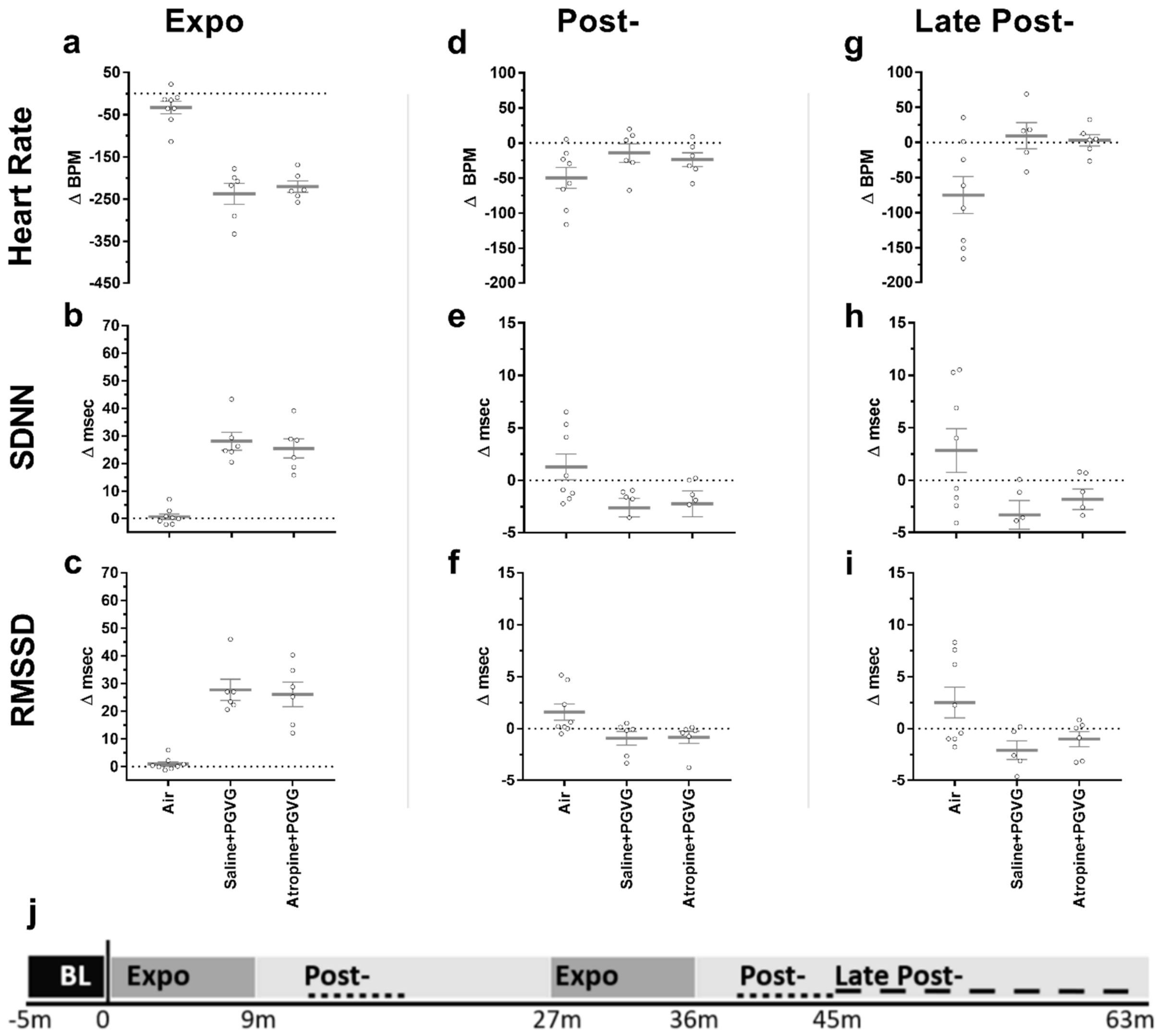

Supplementary Figure 9. Effects of MCS or Acrolein Exposures on Heart Rate and HRV. Individual subject average changes from 5-min baseline in heart rate and HRV (SDNN and RMSSD), stratified by phase, with observed simple means and standard errors indicated by gray bar ± error bars (**a-i**). Phases are indicated by study timeline below, **j**, with Expo denoting puff sessions, Post- denoting 4-9 min after each puff session, and Late Post- indicating 9-28 min after the final puff session (n=6-8/exposure). For least square means and statistical comparisons, see **Fig. 7**.

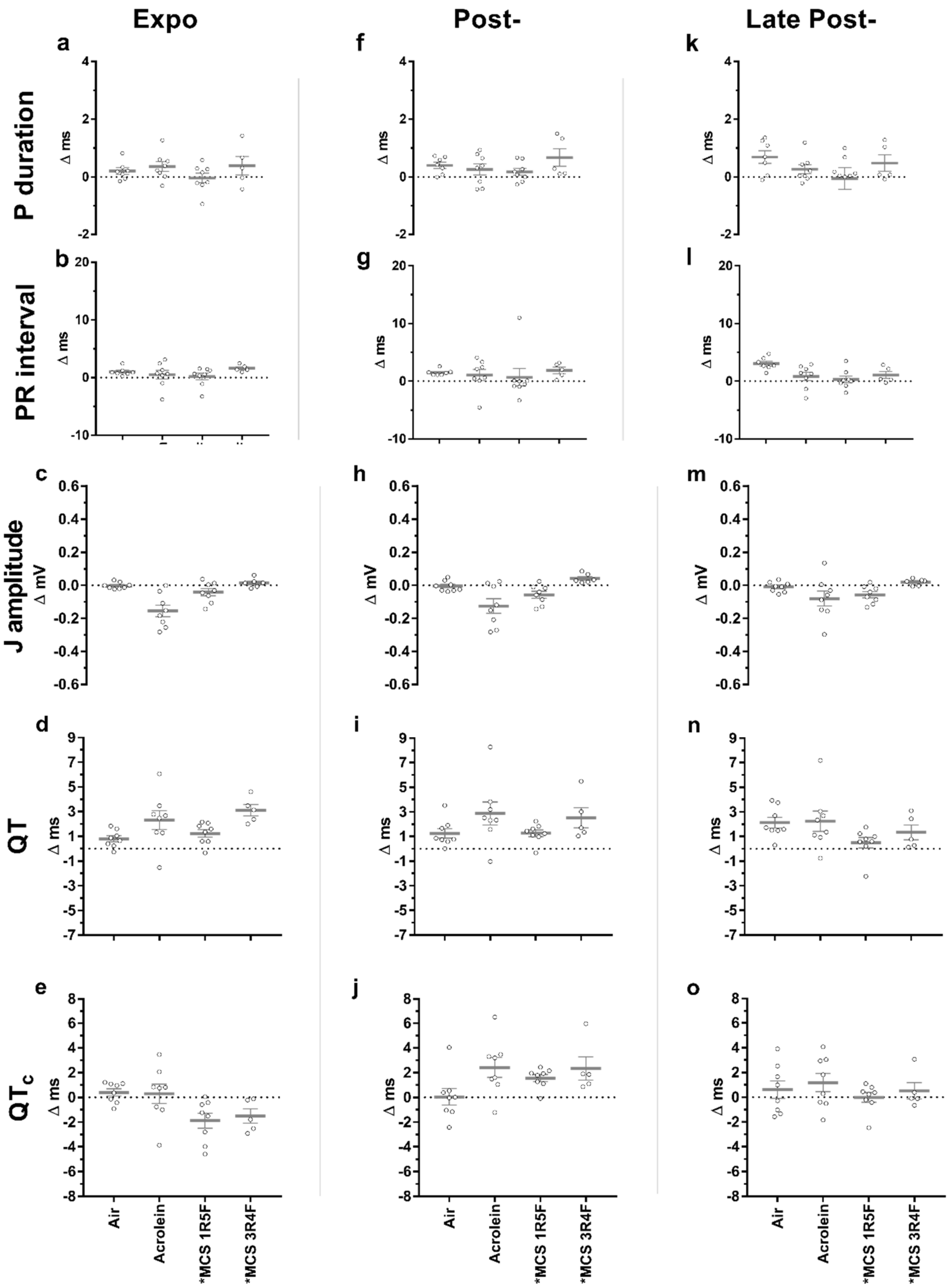

Supplementary Figure 10. Effects of MCS or Acrolein Exposures on ECG Morphology. Individual subject average changes from 5-min baseline in ECG morphologic parameters, stratified by phase, with observed simple means and standard errors indicated by gray bar ± error bars. For least square means and statistical comparisons, see **Fig. 8**.

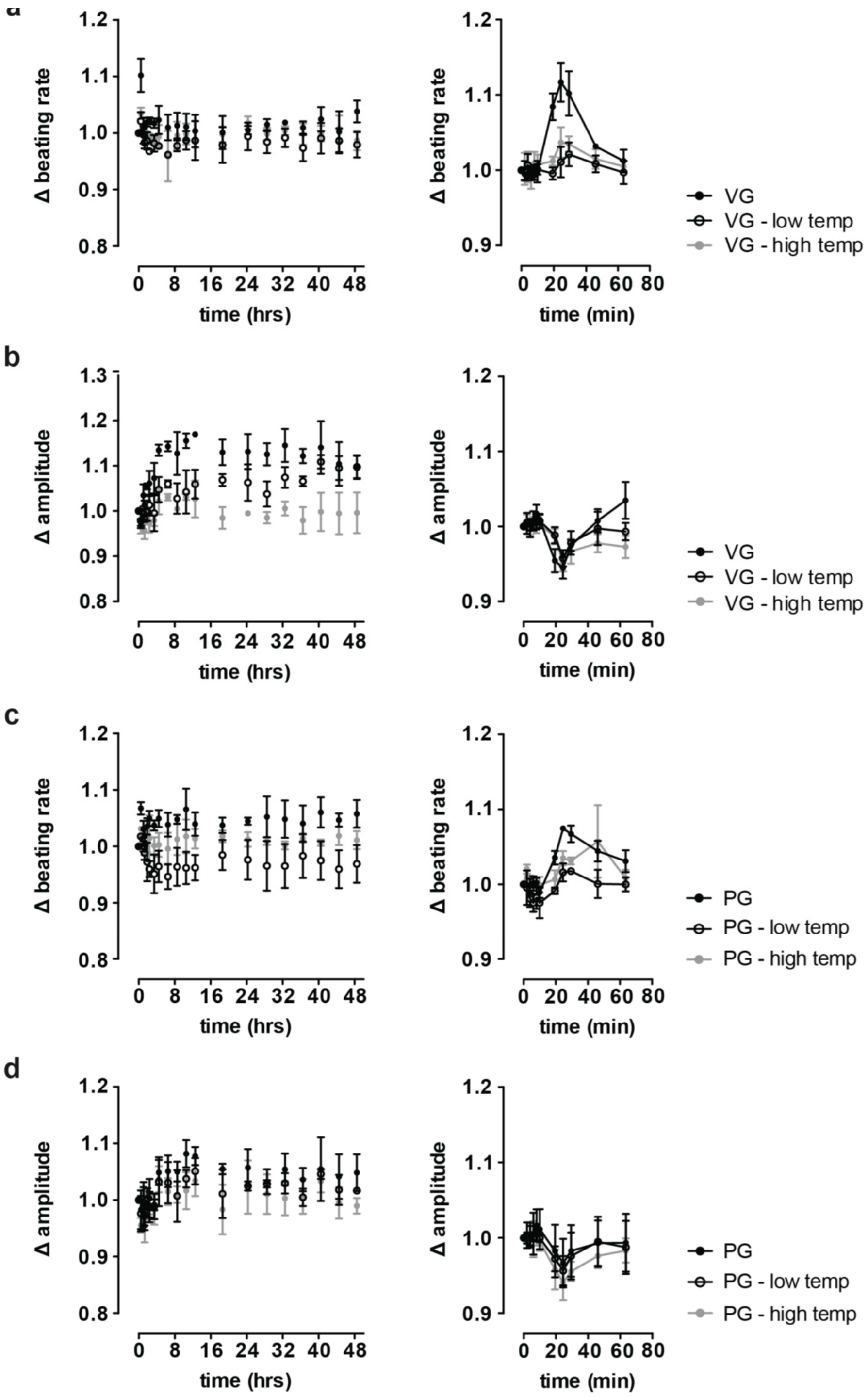

Supplementary Figure 11: Effect of VG and PG on hiPSC-CM beat rate and contractility. Effect of vegetable glycerin (VG; 100 μM; **a,b**) and propylene glycol (PG; 100 μM; **c,d**) and their corresponding thermal products (low temp: 200°C; high temp: 700°C) on hiPSC-CM beat rate and contractile amplitude. Compounds were applied at t = 0 and 24 h and effects are expressed as normalized responses relative to beating rates and amplitudes in control wells containing medium only (-PG/VG) for the same period. Values as means ± SD, with significance determined by two-sided $P < 0.05$ for interaction (unheated vs. both heated forms) in two-way ANOVA.

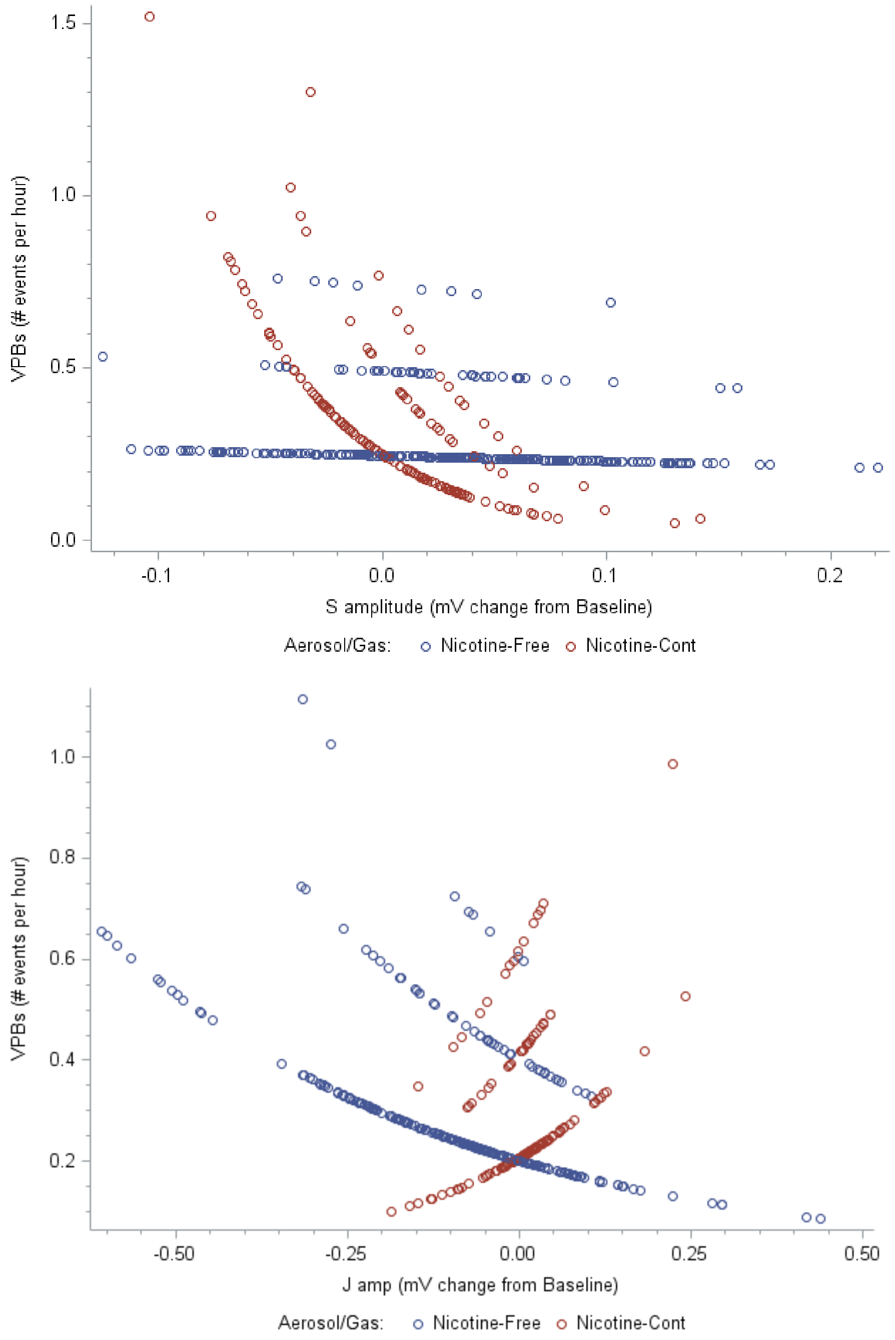

Supplementary Figure 12. Relationship of changes in S amplitude and J amplitude with number of VPBs per hour across exposure phases. Log-linear plots, delineated by the presence (red) or absence (blue) of nicotine in aerosol exposures. VPB estimates (delineated by puff session) were normalized by phase duration (per hour). Data from Air exposures were excluded from these analyses. Line separations occur due to discrete values of VPBs (e.g, 1-3). Nicotine-Cont: nicotine-containing.

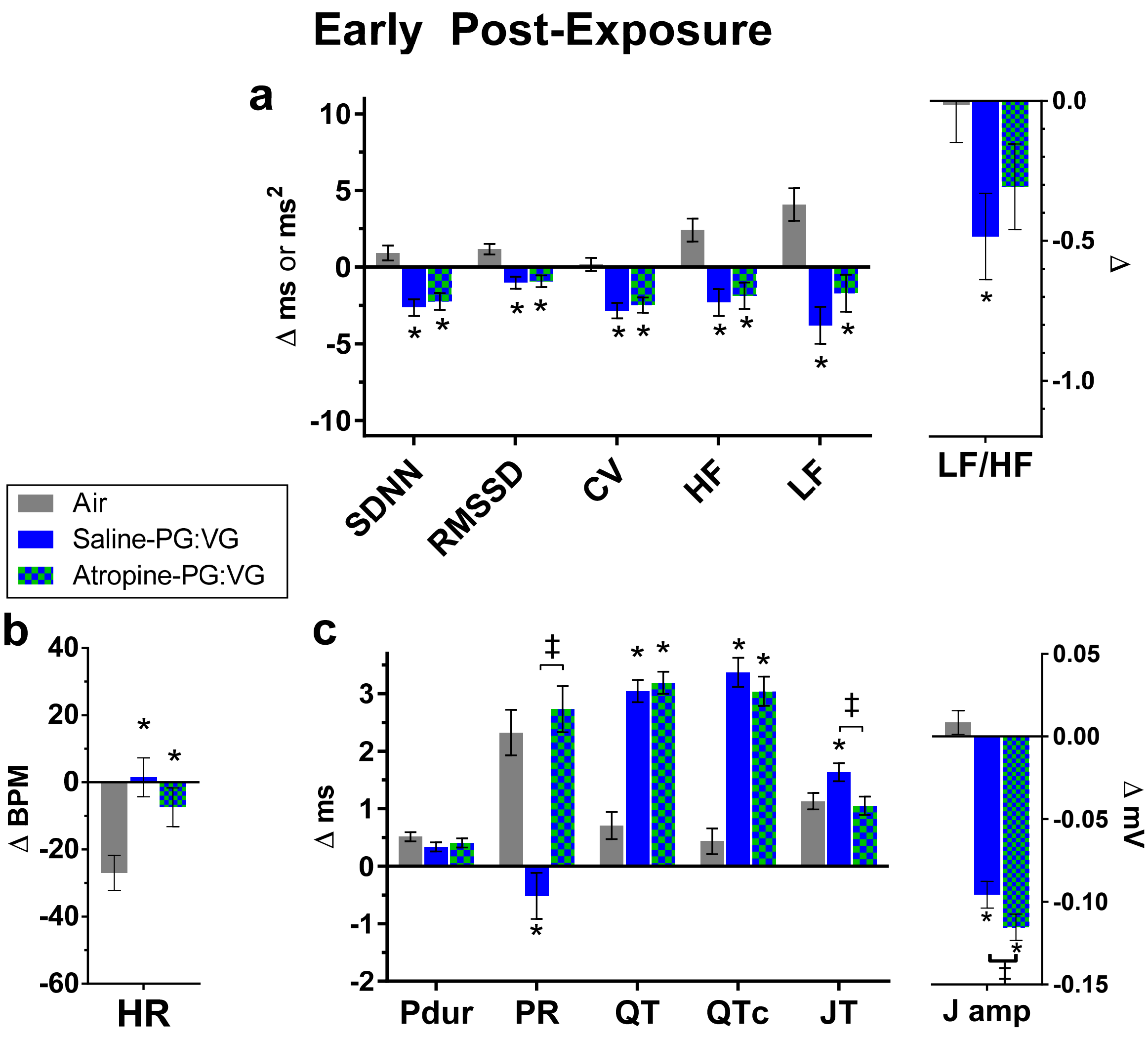

Supplementary Figure 13. During Post-Exposure Phase, Muscarinic Receptor Blockade Alters Electrophysiologic Effects of E-cig Aerosols. Graphs represent least square means (± SEM) changes from 5-min baseline at 4-9 min after each puff session. Significance determined by two-sided P < 0.05 (vs. Air: asterisk; atropine-PG:VG vs. Saline-PG:VG: two-barred cross) in mixed model analyses. Pdur indicates P duration and J amp indicates J amplitude. For averages of individual subjects by phase, and simple means and standard errors, see *Supplementary Figs. 15-17*.

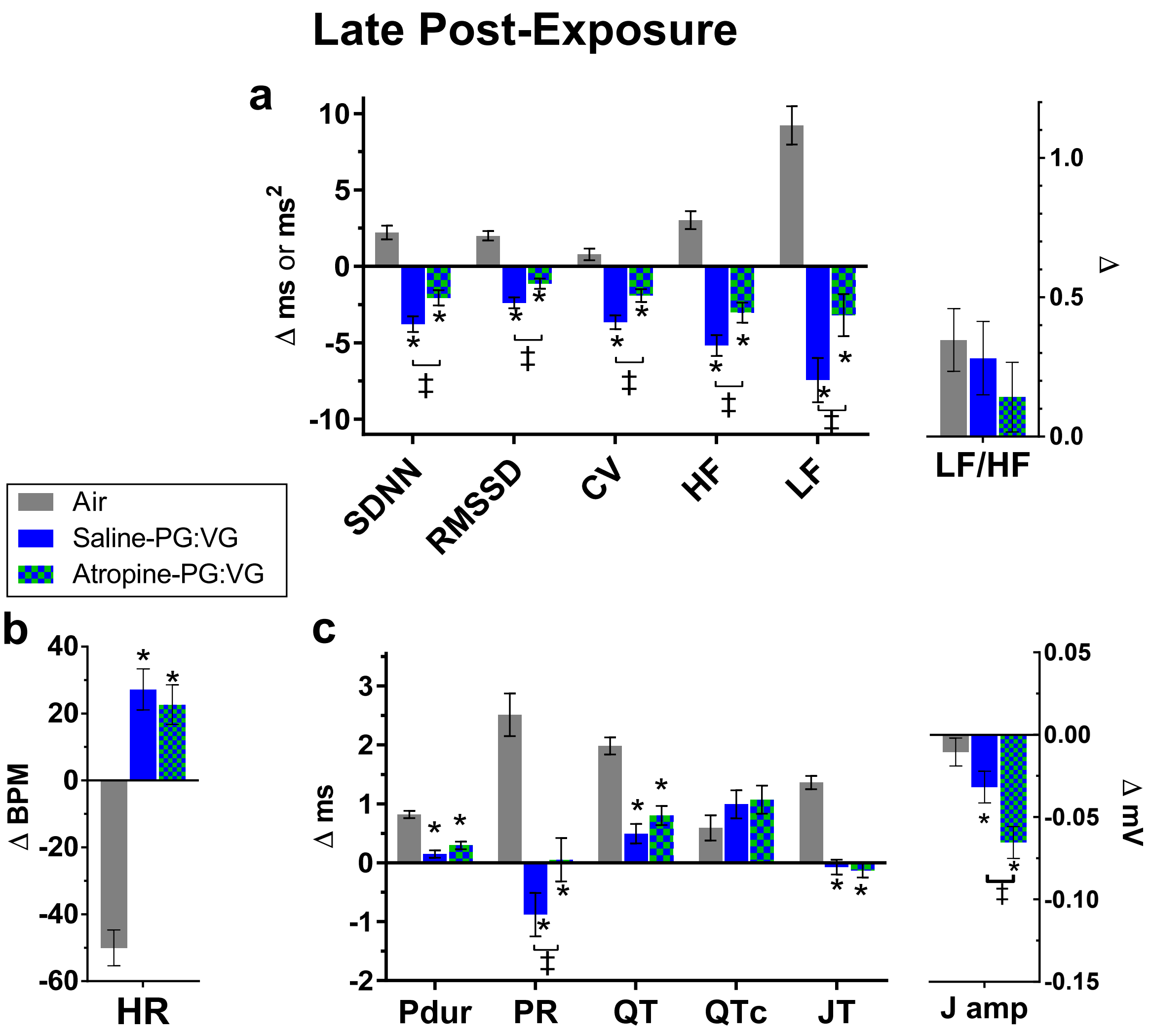

Figure S14. Muscarinic Receptor Blockade Alters Electrophysiologic and Autonomic Effects of E-cig Aerosols Late After Exposure. Graphs represent least square means (± SEM) changes from 5-min baseline at 9-27 min after the final puff session. Significance determined by two-sided P < 0.05 (vs. Air: asterisk; atropine-PG:VG vs. Saline-PG:VG: two-barred cross) in mixed model analyses. Air n=8, Saline + PG:VG n=6, Atropine + PG:VG n=6. Pdur indicates P duration and J amp indicates J amplitude. For averages of individual subjects by phase, and simple means and standard errors, see *Supplementary Figs. 15-17*.

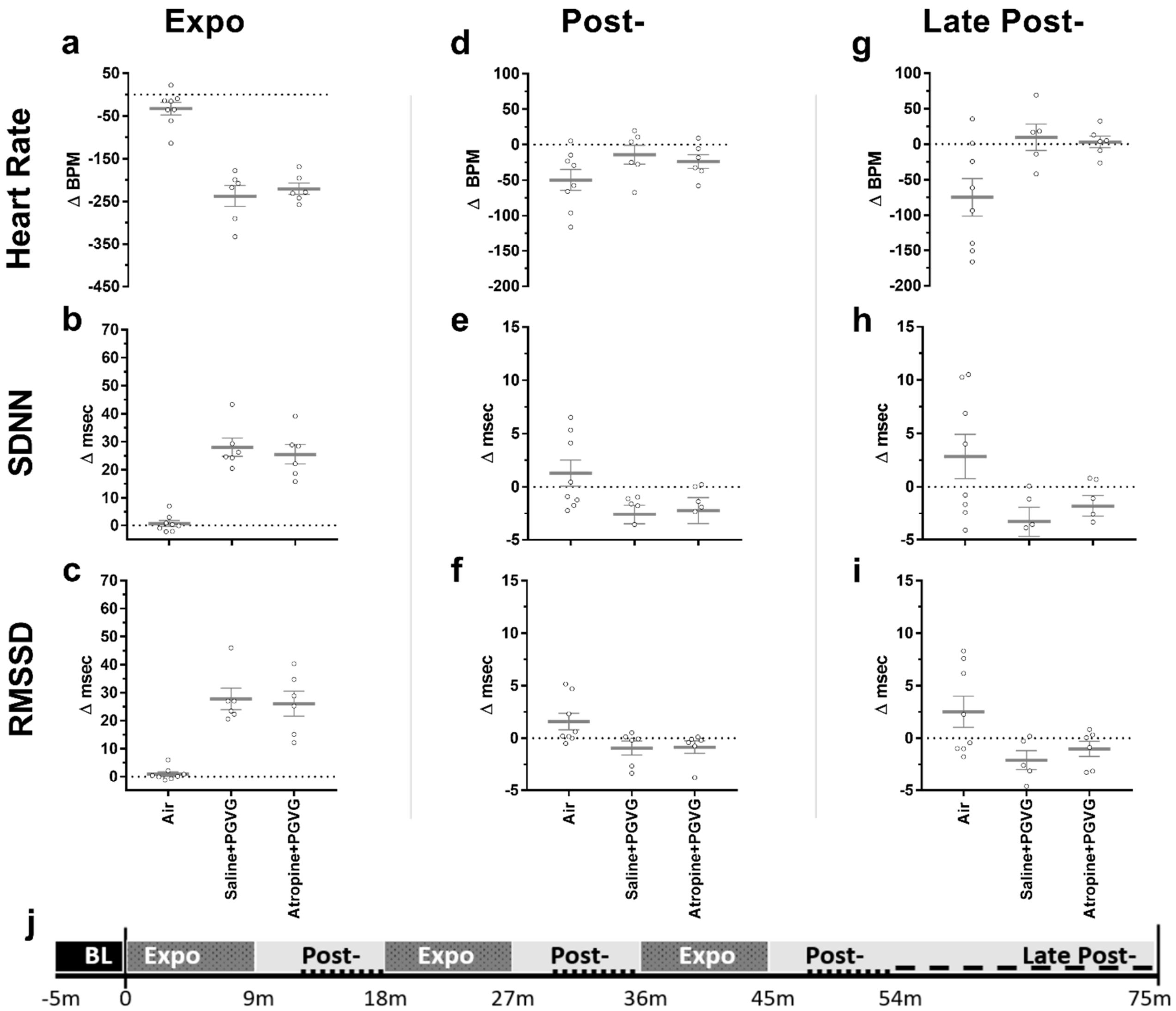

Figure S15. Impacts of Muscarinic Receptor Blockade on Heart Rate and Heart Rate Variability Effects of E-cig Solvent Aerosols. Individual subject average changes from 5-min baseline in heart rate and HRV (SDNN and RMSSD), stratified by phase, with observed simple means and standard errors indicated by gray bar ± error bars (**a-i**). Phases are indicated by study timeline below, **j**, with Expo denoting puff sessions, Post- denoting 4-9 min after each puff session, and Late Post- indicating 9-28 min after the final puff session. Air n=8, Saline + PG:VG n=6, Atropine + PG:VG n=6. For least square means and statistical comparisons, see **Fig. 9** and *Supplementary Figs. 13 and 14*.

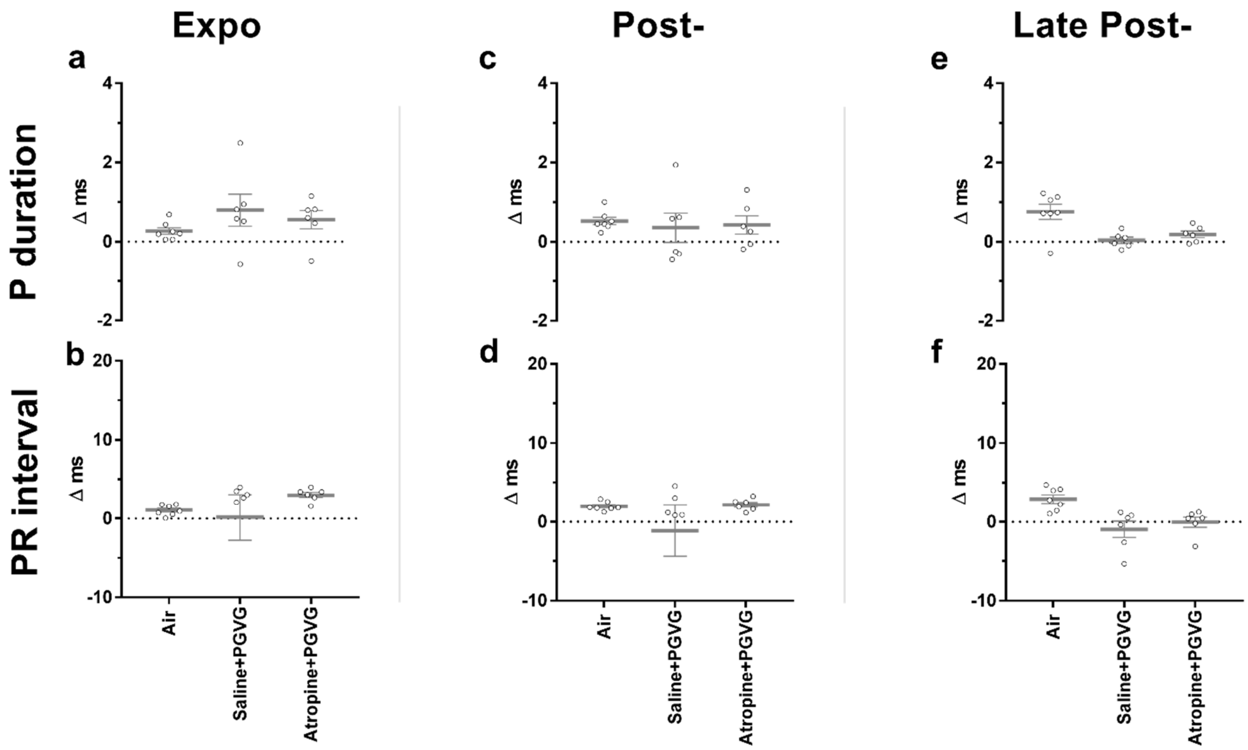

Figure S16. Impacts of Muscarinic Receptor Blockade on Effects of E-cig Solvent Aerosols on Atrial and Atrioventricular Conduction. Individual subject average changes from 5-min baseline in ECG morphology, stratified by phase, with observed simple means and standard errors indicated by gray bar ± error bars (**a-l**). Air n=8, Saline + PG:VG n=6, Atropine + PG:VG n=6. For least square means and statistical comparisons, see **Fig. 9** and *Supplementary Figs. 13 and 14*.

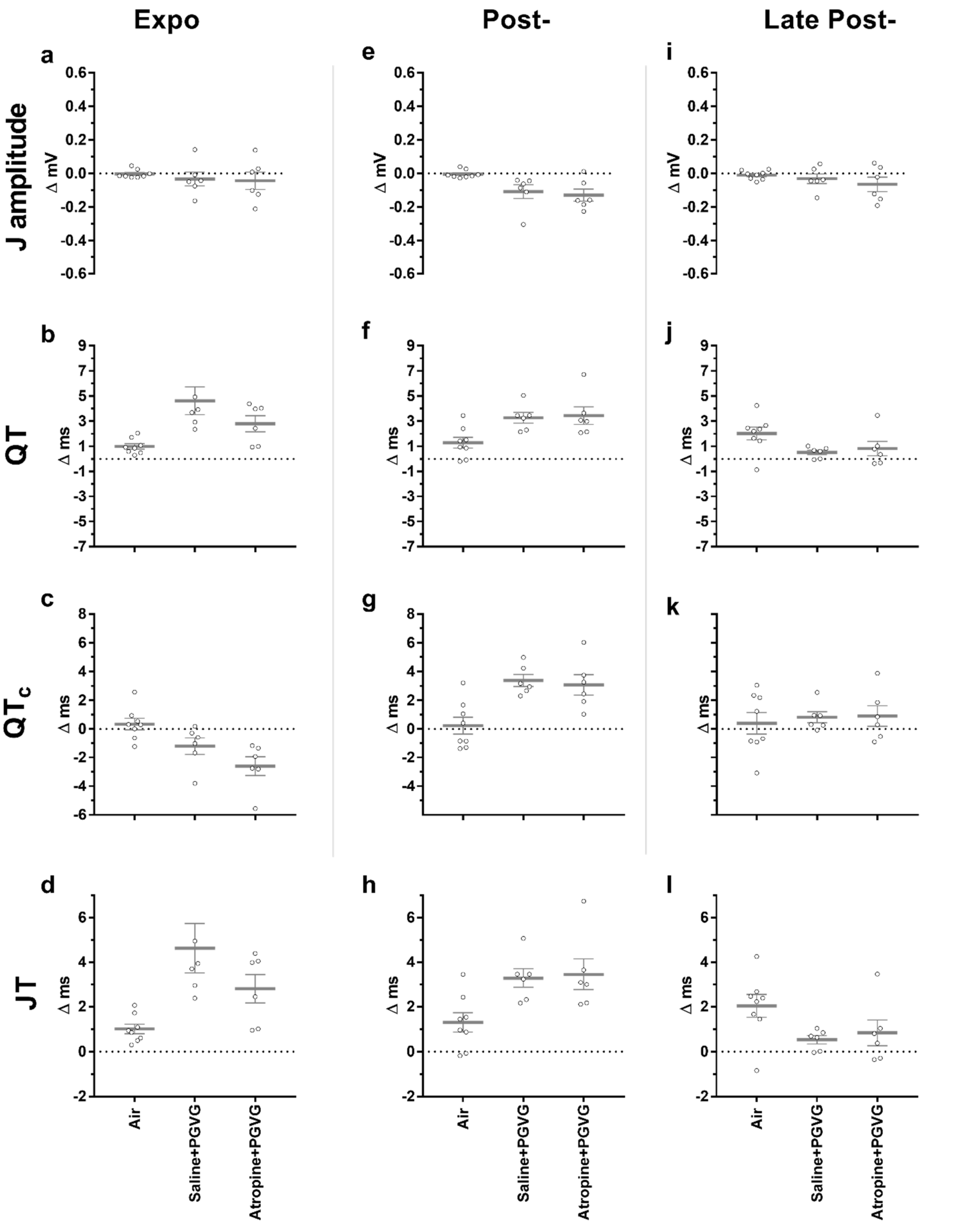

Figure S17. Impacts of Muscarinic Receptor Blockade on Effects of E-cig Solvent Aerosols on Ventricular Repolarization. Individual subject average changes from 5-min baseline in ECG morphology, stratified by phase, with observed simple means and standard errors indicated by gray bar ± error bars (**a-l**). Air n=8, Saline + PG:VG n=6, Atropine + PG:VG n=6. For least square means and statistical comparisons, see **Fig. 9** and *Supplementary Figs. 13 and 14.*

Supplementary References

1. Mitchell GF, Jeron A, Koren G. Measurement of heart rate and q-t interval in the conscious mouse. *Am J Physiol*. 1998;274:H747-751
